# Quantitative Assessment of Nucleocytoplasmic Large DNA Virus and Host Interactions Predicted by Co-occurrence Analyses

**DOI:** 10.1128/mSphere.01298-20

**Published:** 2021-04-21

**Authors:** Lingjie Meng, Hisashi Endo, Romain Blanc-Mathieu, Samuel Chaffron, Rodrigo Hernández-Velázquez, Hiroto Kaneko, Hiroyuki Ogata

**Affiliations:** a Bioinformatics Center, Institute for Chemical Research, Kyoto University, Gokasho, Uji, Japan; b Laboratoire de Physiologie Cellulaire and Végétale, CEA, University Grenoble Alpes, CNRS, INRA, IRIG, Grenoble, France; c Université de Nantes, CNRS UMR 6004, LS2N, Nantes, France; d Research Federation (FR2022) Tara Océan GO-SEE, Paris, France; e Max Planck Institute for Marine Microbiology, Bremen, Germany; National Institute of Advanced Industrial Science and Technology

**Keywords:** NCLDV, *Tara* Oceans, assessment, co-occurrence, host prediction

## Abstract

Nucleocytoplasmic large DNA viruses (NCLDVs) are highly diverse and abundant in marine environments. However, the knowledge of their hosts is limited because only a few NCLDVs have been isolated so far. Taking advantage of the recent large-scale marine metagenomics census, *in silico* host prediction approaches are expected to fill the gap and further expand our knowledge of virus-host relationships for unknown NCLDVs. In this study, we built co-occurrence networks of NCLDVs and eukaryotic taxa to predict virus-host interactions using *Tara* Oceans sequencing data. Using the positive likelihood ratio to assess the performance of host prediction for NCLDVs, we benchmarked several co-occurrence approaches and demonstrated an increase in the odds ratio of predicting true positive relationships 4-fold compared to random host predictions. To further refine host predictions from high-dimensional co-occurrence networks, we developed a phylogeny-informed filtering method, Taxon Interaction Mapper, and showed it further improved the prediction performance by 12-fold. Finally, we inferred virophage-NCLDV networks to corroborate that co-occurrence approaches are effective for predicting interacting partners of NCLDVs in marine environments.

**IMPORTANCE** NCLDVs can infect a wide range of eukaryotes, although their life cycle is less dependent on hosts compared to other viruses. However, our understanding of NCLDV-host systems is highly limited because few of these viruses have been isolated so far. Co-occurrence information has been assumed to be useful to predict virus-host interactions. In this study, we quantitatively show the effectiveness of co-occurrence inference for NCLDV host prediction. We also improve the prediction performance with a phylogeny-guided method, which leads to a concise list of candidate host lineages for three NCLDV families. Our results underpin the usage of co-occurrence approaches for the metagenomic exploration of the ecology of this diverse group of viruses.

## INTRODUCTION

Nucleocytoplasmic large DNA viruses (NCLDVs) represent a group of double-stranded DNA viruses that belong to the viral phylum *Nucleocytoviricota* (Virus Taxonomy, 2019 release), which was previously referred to as *Megavirales* (1, 2). NCLDVs usually possess diverse gene repertoires (74 to more than 2,000 proteins), large genomes (45 kb to 2.5 Mb), and outsized virions (80 nm to 1.5 μm) (3–5). NCLDVs have high functional autonomy and encode components of replication, transcription, and translation systems (3). Recently, a virus that belongs to a new family of NCLDVs called “Medusaviridae” was found to encode five types of histones (6). The existence of metabolically active viral factories and infectious virophages also indicates that the life cycle of NCLDVs is less dependent on host cells than other viruses (7, 8). To further understand the features of these giant viruses, a first crucial step is to identify their hosts, i.e., “who infects whom?”

NCLDVs are known to infect a broad range of eukaryotes, from unicellular eukaryotes and macroalgae to animals (9, 10). Amoebae are frequently used hosts in coculture to isolate large NCLDVs (11). However, there is growing evidence, especially in marine systems, that NCLDVs can infect many phytoplankton groups, such as Pelagophyceae, Mamiellophyceae, Dinophyceae, and Haptophyte (12–14). Several other nonphotosynthetic eukaryotic lineages, such as Bicoecea and Choanoflagellatea, were also reported as experimentally identified NCLDV hosts in marine environments (15, 16). *Iridoviridae* can also infect marine organisms, from small invertebrates to large vertebrates (17, 18). Together, these studies indicate ubiquitous infectious relationships between NCLDVs and a wide range of marine eukaryotes. However, our understanding of NCLDV-host systems is very limited because few viruses have been isolated so far.

The number of viruses and hosts isolated in the laboratory represents a very small fraction of existing interactions in the ocean. Indeed, NCLDVs have been found to be highly diverse and abundant based on omics data (19, 20). In only a few liters of coastal seawater, more than 5,000 *Mimiviridae* species were detected; by comparison, only 20 *Mimiviridae* with known hosts have been well investigated (21). Global marine metagenomic data have revealed that the richness and phylogenetic diversity of NCLDVs are even higher than those of an entire prokaryotic domain (22). From biogeographical evidence, it is clear that these viruses are prevalent in the marine environment but have a heterogeneous community structure across sizes, depths, and biomes (23). Marine metatranscriptomic data have also shown that NCLDVs are active everywhere in sunlit oceans and may infect hosts from small piconanoplankton (0.8 to 5 μm) to large mesoplankton (180 to 2,000 μm) (24).

Previous studies also demonstrated that NCLDVs have the potential to infect a greater diversity of hosts than known to date through gene transfer analyses (25, 26). NCLDVs might have started coevolving with eukaryotes even before the last eukaryotic common ancestor (LECA) (27). A recent study supported this hypothesis by showing that some NCLDVs encode viractins (actin-related genes in viruses), which could have been acquired from proto-eukaryotes and possibly reintroduced in the pre-LECA eukaryotic lineage (28). Together, these findings underline a lack of knowledge about NCLDV biology and host diversity. Therefore, more effort is needed to identify hosts to elucidate the poorly known virus-host relationships and the largely unknown NCLDV world.

Substantial effort has been made to reveal interactions between NCLDVs and their putative hosts. Apart from the coculture method, other culture-independent experimental methods, such as high-throughput cell sorting, are also being used (11, 16). Metagenomics, which is particularly useful to assess a large fraction of ecosystem diversity, has been increasingly used to investigate NCLDVs host range. Comparative genomics analyses, such as the identification of horizontal gene transfer (HGT) predictions, have largely expanded the host range of NCLDV (25, 26). Investigating endogenous NCLDV fragments in certain eukaryotic lineages can also be useful for inferring species-specific virus-host associations (29).

Abundance-based analyses have been used for host prediction and are supposed to be effective because viruses can only thrive in an environment where their hosts exist (19, 30). In addition to virus-host relationships, this strategy has also been used to predict the association between NCLDVs and their “parasites” (virophages) (31). However, the correlation-based prediction is also controversial for viral host prediction since the abundance dynamics of viruses and their hosts (e.g., *Emiliania huxleyi* and Heterosigma akashiwo viruses) are sometimes not concordant (32, 33). Usually, validation with known virus-host relationships or corroboration with genomic evidence (e.g., HGT) is used to assess network-based predictions (19, 30). However, the effectiveness of previous and novel co-occurrence network methods has never been quantitatively tested for NCLDV host prediction. The current lack of quantitative assessment hinders the widespread use of this approach. Therefore, dedicated methods are needed to test the accuracy of NCLDV host prediction with co-occurrence networks and to improve the performance of co-occurrence-based predictions.

The *Tara* Oceans expedition is a global-scale survey on marine ecosystems that expands our knowledge of microbial diversity, organismal interactions, and ecological drivers of community structure (34). The present study used *Tara* Oceans metagenomic and metabarcoding data sets to predict virus-host relationships between NCLDVs and eukaryotes by constructing co-occurrence networks using different methods. To quantitatively assess the performance of network-based host prediction, we employed the positive likelihood ratio (LR+) using reference data for known NCLDV-host relationships. We developed a phylogeny-based enrichment analysis approach, Taxon Interaction Mapper (TIM), to enhance the performance in detecting positive signals in the intricate inferred networks. TIM has previously been used for viruses with high importance in predicting the carbon export efficiency (35), but without a quantitative assessment of its effectiveness. In this study, we assessed the performance of TIM as a filter of co-occurrence networks. We examined NCLDV-virophage networks, which further justify the use of co-occurrence and filtering approaches to identify NCLDV interaction partners.

## RESULTS

### NCLDV-eukaryote co-occurrence networks.

**FIG 1:**
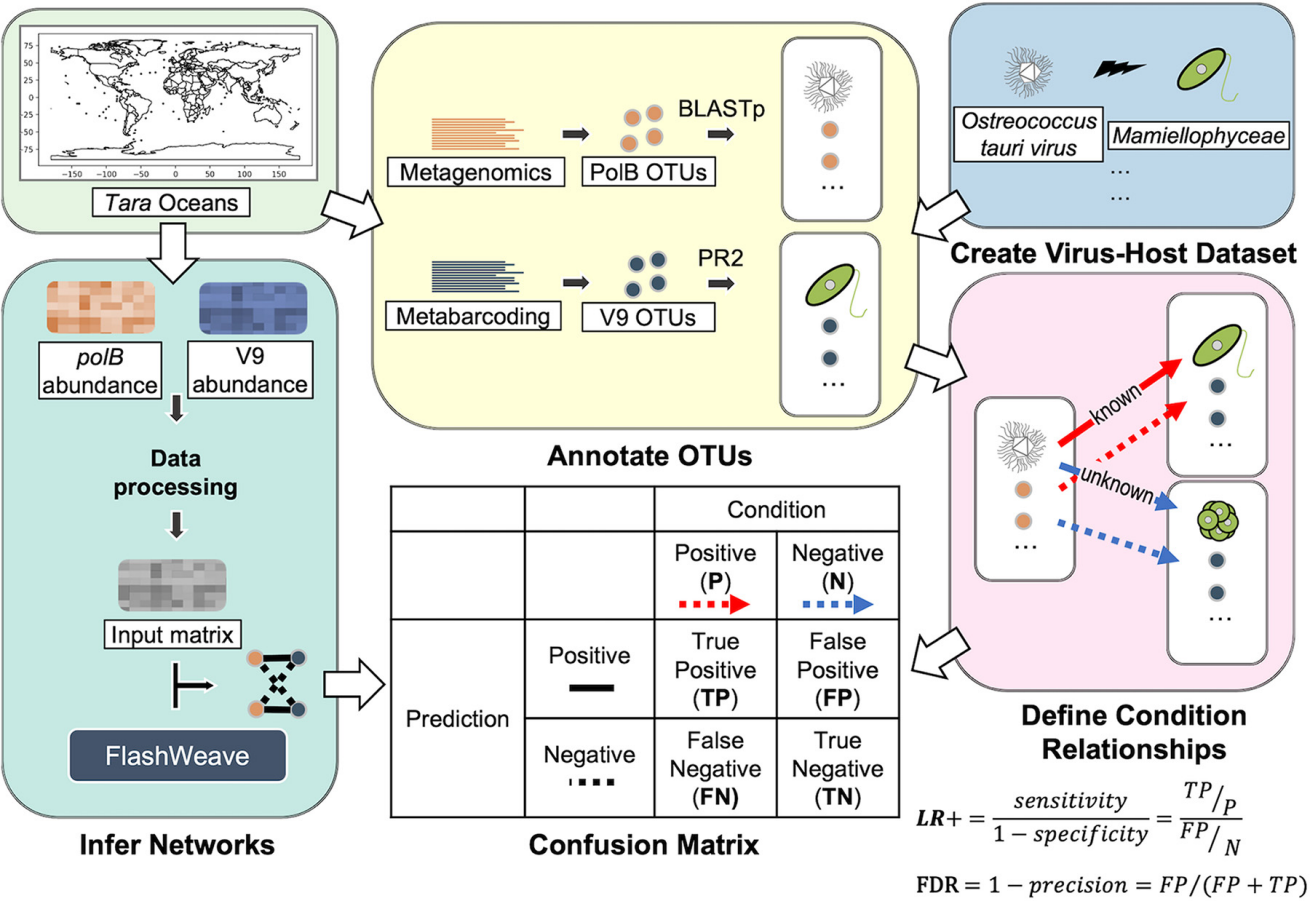
Overall workflow for inferring co-occurrence networks and quantitative assessment. This figure shows how the input data (*Tara* Oceans metagenomics and metabarcoding data) were used in this study. The definition of the confusion matrix for quantitative assessment is shown in the table. The LR+ and FDR equations are given at the lower right corner of the plot.

**FIG 2:**
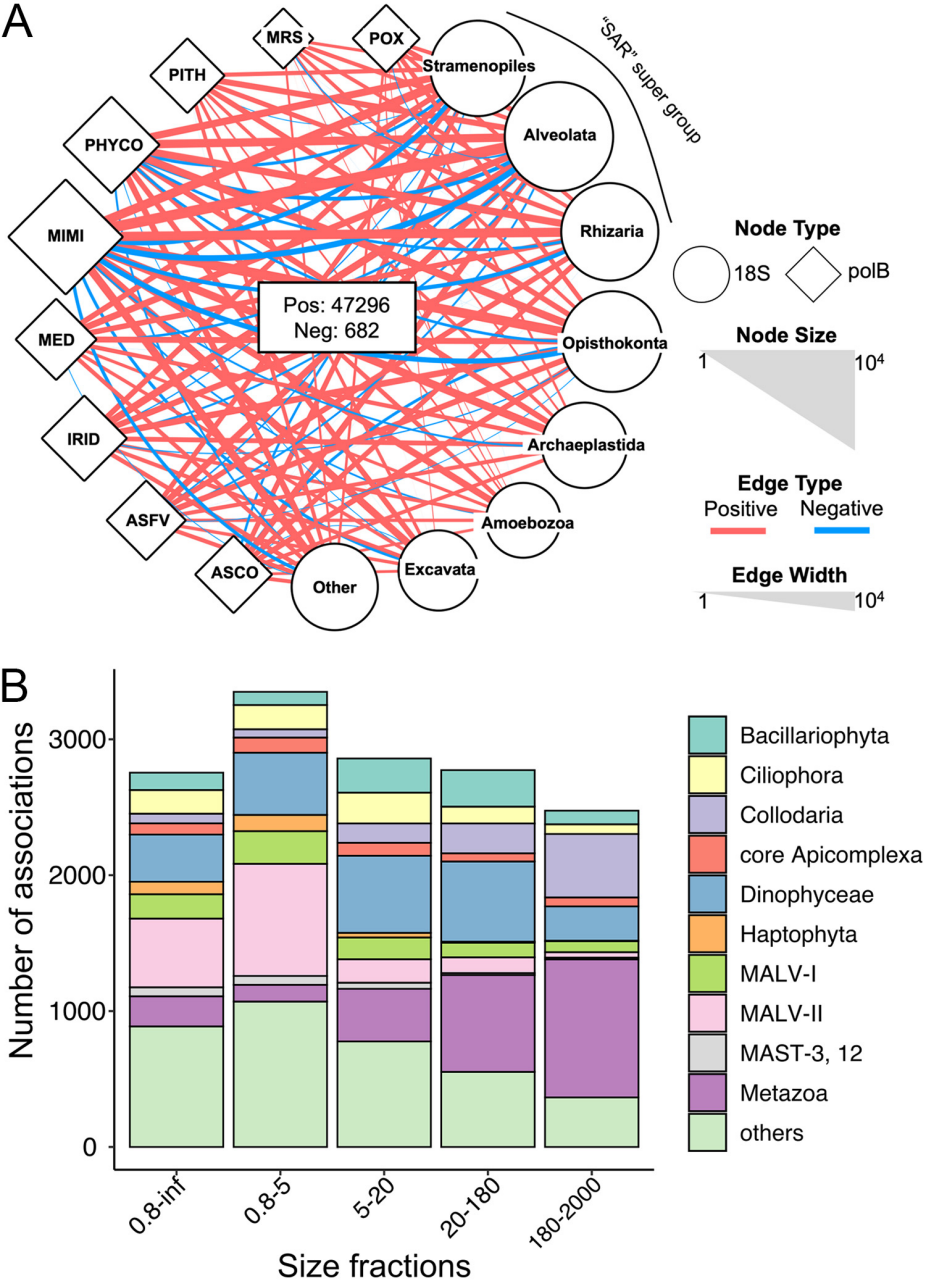
*polB*-V9 co-occurrence network. (A) We performed co-occurrence analysis at the OTU level and constructed the network with pooled *polB*-V9 associations from five size fraction networks. To better display co-occurrence patterns, PolB OTUs were grouped at the family or family-like level, and V9 OTUs were grouped using annotation at high taxonomic ranks. The size of each node indicates the number of OTUs that belong to the group, and the width of each edge indicates the number of associations between two connected groups. Associations with positive weight are shown in red and negative associations are shown in blue. (B) Number of associations connected to NCLDVs for each major eukaryotic lineage in five size fractions. The top 10 lineages were retained, and other lineages were omitted and shown as “others.” Size fractions are presented in μm.

From the data sets that corresponded to five size fractions (see Fig. S1 in the supplemental material), we generated five co-occurrence networks on a global scale (Fig. 1; see also Fig. S2A). Altogether, these networks were composed of 20,148 V9 and 5,234 *polB* operational taxonomic units (OTUs) (nodes) connected by 47,978 *polB*-V9 associations (edges). A total of 47,296 associations had positive weights, and 682 associations had negative weights (Fig. 2A). The associations that involved the family *Mimiviridae* were numerically dominant (*n *=* *36,830). *Marseilleviridae*, forming the least associations in the networks, had 132 edges with eukaryotes. Taxonomic annotation of eukaryotic OTUs indicated that Alveolata, Opisthokonta, Rhizaria, and Stramenopiles were the major four eukaryotic groups connected to NCLDVs (with 21,167, 9,179, 6,521, and 5,327 edges, respectively). Three of these eukaryotic groups belong to the SAR supergroup (i.e., Stramenopiles, Alveolata, and Rhizaria), which represented 68.81% of the total associations. Regarding the pairs between viral families and eukaryotic lineages, *Mimiviridae* and Alveolata showed the largest number of edges (*n *=* *16,548). Besides NCLDV-eukaryote associations, we detected 57,495 *polB-polB* associations and 234,448 V9-V9 associations (see Fig. S2B). We also included environmental parameters in the network inference and identified 25 pairs of associations between environmental parameters and *polB* OTUs (see Table S1 in the supplemental material).

10.1128/mSphere.01298-20.1 FIG S1 Stations, samples, and OTUs used in this study. The *Tara* Oceans expedition is an international and multidisciplinary project; the stations are distributed worldwide. (A) After dataset treatment, the samples from a total of 84 stations were used in this study, and the retained stations were evenly distributed in the ocean. (B) Depending on the individual size fractions, 84 to 127 samples were retained and included in the co-occurrence analysis. (C) Numbers of OTUs (V9 and *polB* are presented separately) in the final five individual size fraction matrices. Size fractions are presented in μm. (D and E) The Shannon diversity (D) and richness index (E) were calculated for eukaryotic communities, from small piconanoplanktons (0.8 to 5 μm) to large mesoplanktons (180 to 2,000 μm). Only the samples involved in co-occurrence inference for host prediction were used. Box plots are shown to summarize variation in richness and Shannon’s index across (centerline, median; box limits, 25 to 75% quantiles). Download FIG S1, TIF file, 0.3 MB. Copyright © 2021 Meng et al. 2021 Meng et al. https://creativecommons.org/licenses/by/4.0/ This content is distributed under the terms of the Creative Commons Attribution 4.0 International license.

10.1128/mSphere.01298-20.2 FIG S2 Co-occurrence networks inferred using FlashWeave. The size of each node indicates the number of OTUs that belong to the group, and the width of each edge indicates the number of associations between two connected groups. Associations with positive weight are shown in red and negative associations are shown in blue. (A) *polB*-V9 co-occurrence networks of four individual size fractions and a broad range of size fractions (0.8–inf μm). (B) Networks consist of *polB*-*polB* and V9-V9 associations. Both associations were pooled from five size fraction networks. Size fractions are presented in μm. Download FIG S2, TIF file, 1.1 MB. Copyright © 2021 Meng et al. 2021 Meng et al. https://creativecommons.org/licenses/by/4.0/ This content is distributed under the terms of the Creative Commons Attribution 4.0 International license.

10.1128/mSphere.01298-20.8 TABLE S1 Associations between NCLDVs and environmental variables inferred by FlashWeave. Download Table S1, DOCX file, 0.07 MB. Copyright © 2021 Meng et al. 2021 Meng et al. https://creativecommons.org/licenses/by/4.0/ This content is distributed under the terms of the Creative Commons Attribution 4.0 International license.

The number of NCLDV-eukaryote associations generally decreased with enlarging size fraction (see Fig. S2A). The largest number of *polB-*V9 associations were found in the 0.8- to 5-μm fraction (*n *=* *10,647). Correspondingly, the eukaryotic community in the 0.8- to 5-μm fraction had the greatest diversity (see Fig. S1D and E). However, the ≥0.8 μm (referred to here as 0.8–inf μm) size fraction network was the largest (*n *=* *10,477) for edges with positive weights. With the annotation of major lineages, the eukaryotic community compositions in the networks varied across different size fractions (Fig. 2B). In the smallest size fraction (0.8 to 5 μm) and the large range size fraction (0.8–inf μm), Marine Alveolate Group II was the eukaryotic lineage with the largest number of associations with NCLDVs (21.39 and 19.98%, respectively). Dinophyceae was the second largest group connected to NCLDVs in these two size fractions and showed the largest number of connections with NCLDVs in the 5- to 20-μm network (22.22% of total interactions). The viral associations with Metazoa and Collodaria increased with increasing size fractions. In the largest 180- to 2,000-μm size fraction network, Metazoa contributed 39.31% of the total *polB*-V9 edges.

**FIG 3:**
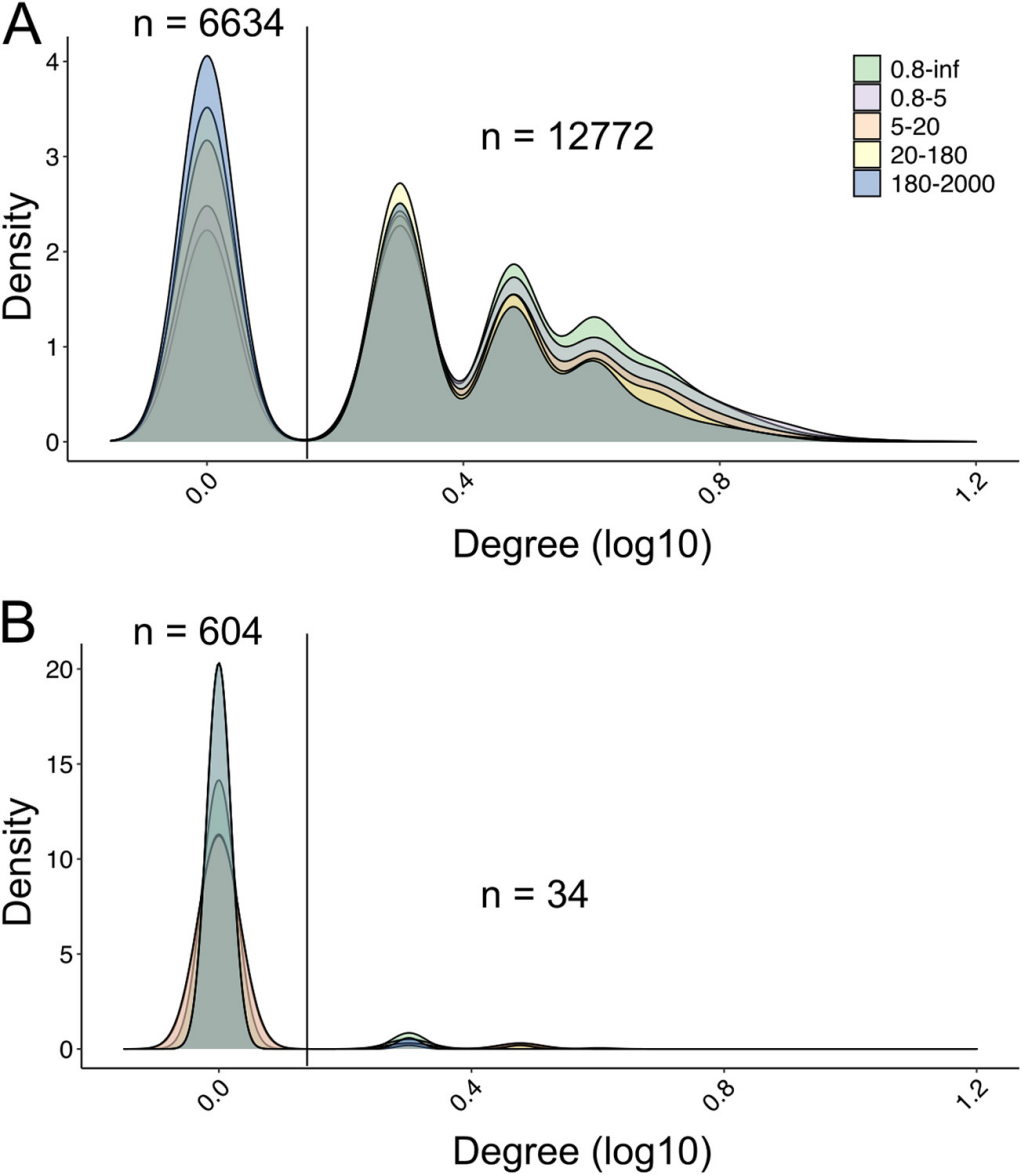
Density plots for the degree of NCLDV nodes in co-occurrence networks. The degree of an NCLDV node is given by the associations between this node and eukaryotes in the networks. The numbers of NCLDV nodes are given at the top of the density values. (A) Positive degree (number of positive associations per node) for NCLDV nodes in five size fraction networks. (B) Negative degree (number of negative associations per node) for NCLDV nodes in five size fraction networks. Size fractions are presented in μm. NCLDV nodes with degree = 1 and degree > 1 are separated using a vertical line, and the number of nodes is given.

We calculated the degree of nodes (number of connected edges) for each NCLDV *polB* OTU (Fig. 3A and B). Naturally, the average degree of positive associations per *polB* was higher than negative edges in all size fractions and decreased along with increasing size fractions (2.69, 2.40, 2.25, and 2.10 from 0.8 to 5 μm to 180 to 2,000 μm and 2.76 for 0.8–inf). Most of the *polB* nodes had more than one positive association (Fig. 3A). Together with the taxonomic annotation of nodes, *polB*-V9 associations in the networks generated with the *Tara* Oceans data revealed their high dimensionality and complexity.

### Network validation.

**FIG 4:**
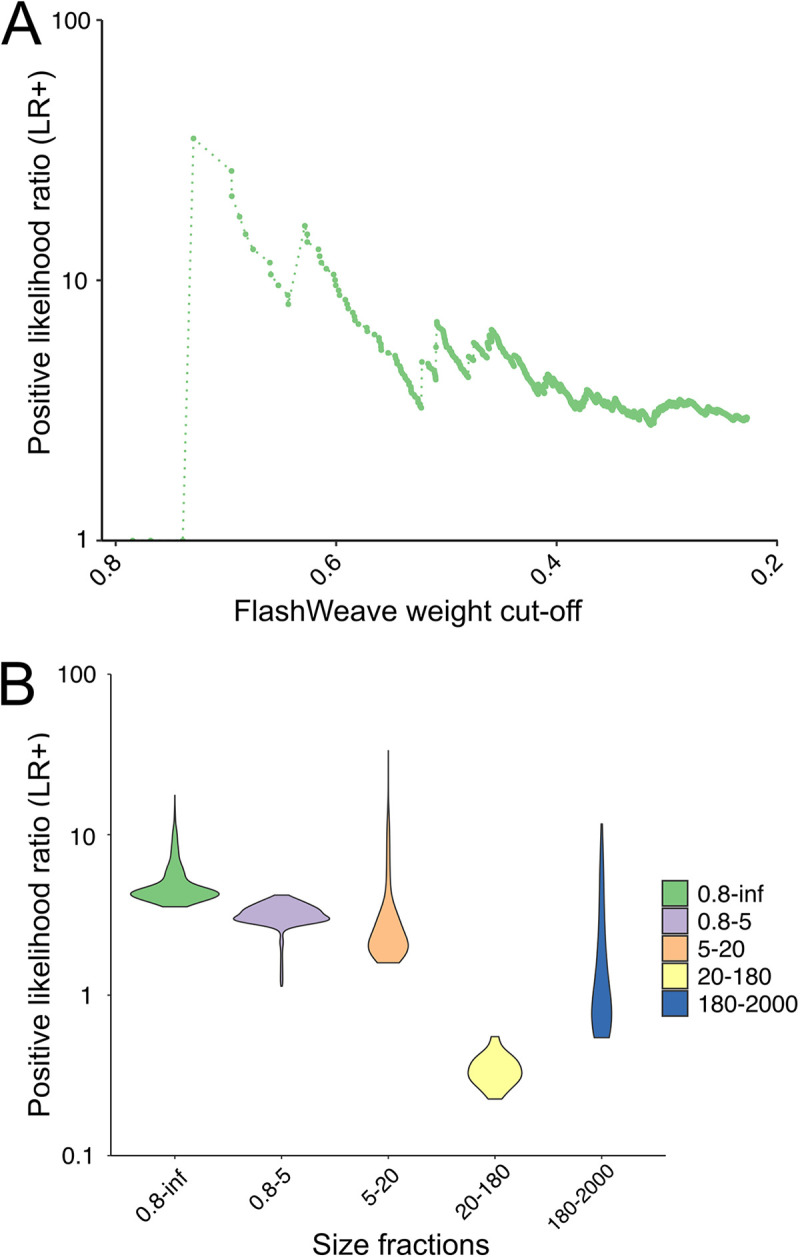
Positive likelihood ratios (LR+) in the NCLDV virus-host validation. (A) General performance of co-occurrence networks is shown with the LR+ calculated with associations pooled from five size fractions networks. To show the relationship between LR+ and FlashWeave association weight, the LR+ values are plotted by dots and connected by a dashed line along with the association weight. (B) Performance of each size fraction network is shown with the violin plot by ggplot2 with a bandwidth of 2. Size fractions are presented in μm.

We quantitatively assessed the performance of predicting *polB*-V9 associations using the positive likelihood ratio (LR+) (Fig. 1 and 4; see also Fig. S4). By defining groups of metagenomic PolBs as described in Materials and Methods, 932 OTUs were recruited in the validation, and these sequences contributed 6191 *polB*-V9 associations in the FlashWeave networks (see Fig. S3). To obtain an overall performance, we assessed the pooled associations (4,069 associations after removing redundancy; see Fig. S4D) from the five co-occurrence networks. LR+ was separately calculated for edges with positive and negative weights because they may represent different infectious patterns. As shown in Fig. 4A, the LR+ of host prediction for positive associations was higher than 1 (LR+ = 1 indicates no change in the likelihood of the condition), and generally increased with the cutoff for FlashWeave weights. In high-weight regions: (i) weight > 0.6, the LR+ of associations was higher than 10; and (ii) weight > 0.4, the LR+ was roughly higher than 4. Nonetheless, the false discovery rate (FDR) was high (see Fig. S4A, true- and false-positive rates are given in Fig. S4C), which indicated that the predictions contained numerous virus-host edges that were not considered condition positive. The FDRs were 91.67 and 96.34% when the weight cutoffs were 0.6 and 0.4, respectively. There were no known NCLDV-host pairs found in the negative networks (see Fig. S4B). The analysis of the remaining part of our study was thus conducted for positive associations.

10.1128/mSphere.01298-20.3 FIG S3 Number of environmental PolB OTUs recruited in the network validation. The 932 OTUs were grouped using 69 reference NCLDV sequences. Apart from *Phycodnaviridae* and *Mimiviridae*, other families did not recruit many environmental OTUs; these families are shown together under an additional group (“Others”). Download FIG S3, TIF file, 0.2 MB. Copyright © 2021 Meng et al. 2021 Meng et al. https://creativecommons.org/licenses/by/4.0/ This content is distributed under the terms of the Creative Commons Attribution 4.0 International license.

10.1128/mSphere.01298-20.4 FIG S4 Performance of networks in the validation of NCLDV virus-host prediction. (A) FDR of the co-occurrence network, which consists of positive associations pooled from five size fractions networks. (B) Positive likelihood ratio (LR+) of the co-occurrence network, which consists of negative weight associations pooled from five size fractions networks. (C) Positive rate of the co-occurrence network, which consists of positive associations pooled from five size fractions networks. True positive rate (sensitivity, purple) and false-positive rate (green) are given. (D) Number of predicted *polB*-V9 positive associations of the co-occurrence network. The values are plotted by dots and connected by a dashed line along with the association weight. Download FIG S4, TIF file, 0.1 MB. Copyright © 2021 Meng et al. 2021 Meng et al. https://creativecommons.org/licenses/by/4.0/ This content is distributed under the terms of the Creative Commons Attribution 4.0 International license.

Comparing the performance between different size fractions indicated that the networks of small size fractions (including the 0.8–inf-μm size fraction) performed better in predicting the NCLDV-host relationships (Fig. 4B; see also Fig. S5). The 0.8–inf-μm size fraction had the highest average LR+ out of the five size fractions (LR+ = 4.97). The LR+ of small size fractions was generally higher than that of large size fractions, but there were exceptions between 180 to 2,000 μm and 20 to 180 μm. The LR+ of the associations in the 0.8–inf-μm, 0.8- to 5-μm, and 5- to 20-μm fractions was greater than 1. Different from the average results, when the weight is greater than 0.8, the associations of the 5- to 20-μm size fraction had the best performance in terms of both LR+ and FDR (see Fig. S5A and B).

10.1128/mSphere.01298-20.5 FIG S5 Comparison of different size fraction networks in the validation of NCLDV virus-host prediction. (A) Positive likelihood ratio (LR+) of co-occurrence networks of five individual size fraction networks. (B) FDR of co-occurrence networks of five individual size fractions networks. (C) Sensitivity of co-occurrence networks of five individual size fraction networks. (D) Number of predicted *polB*-V9 positive associations of co-occurrence networks of five individual size fraction networks. Size fractions are presented in μm. The values are plotted by dots and connected by a dashed line along with the association weight. Download FIG S5, TIF file, 0.2 MB. Copyright © 2021 Meng et al. 2021 Meng et al. https://creativecommons.org/licenses/by/4.0/ This content is distributed under the terms of the Creative Commons Attribution 4.0 International license.

We also compared abundance filtration strategies using FlashWeave-S (sensitive model) and FlashWeave-HE (heterogeneous model) but did not find a consistent pattern in prediction performance (see Fig. S6A and B). The networks from the Q1 filtration strategy performed best using FlashWeave-S, but Q1 (lower quartile) filtration was not better than Q2 (middle quartile) for FlashWeave-HE inferred networks. FlashWeave-S had a better performance than HE model with any filtration strategy. Finally, we compared the performance of networks inferred by all three methods: FlashWeave-S, FastSpar, and Spearman. Three methods generated a comparable number of positive associations, but FlashWeave-S made the largest number of true positive predictions (see Fig. S6C D).

10.1128/mSphere.01298-20.6 FIG S6 Performance of different input data trimming strategies and network inference methods. To show if the OTU abundance influences the performance of the networks in the NCLDV virus-host validation, the datasets were treated by three quartile filtrations before the network inference, and the positive likelihood ratios (LR+) were compared. “S”, FlashWeave sensitive model, and “HE”, FlashWeave heterogeneous model, were performed separately. (A) Positive likelihood ratio (LR+) of networks inferred through different strategies. The plots are shown with a violin plot constructed by ggplot2 with a bandwidth of 2. (B) The number of inferred positive weight associations through different strategies, the number of *polB*-V9 pairs recruited in the validation is given. The networks were inferred using FastSpar, FlashWeave sensitive model, and Spearman. (C) The positive likelihood ratio (LR+) of the pooled associations of the five size fractions inferred by different methods were compared. To reduce the time of network inference in the large *Tara* Oceans datasets, upper quartile (Q3) trimming was performed on the input data. (D) The number of inferred positive weight associations by three methods, the number of *polB*-V9 pairs recruited in the validation is given (true positives and false positives are shown separately). Download FIG S6, TIF file, 0.2 MB. Copyright © 2021 Meng et al. 2021 Meng et al. https://creativecommons.org/licenses/by/4.0/ This content is distributed under the terms of the Creative Commons Attribution 4.0 International license.

### Assessment of host prediction improvement.

**FIG 5:**
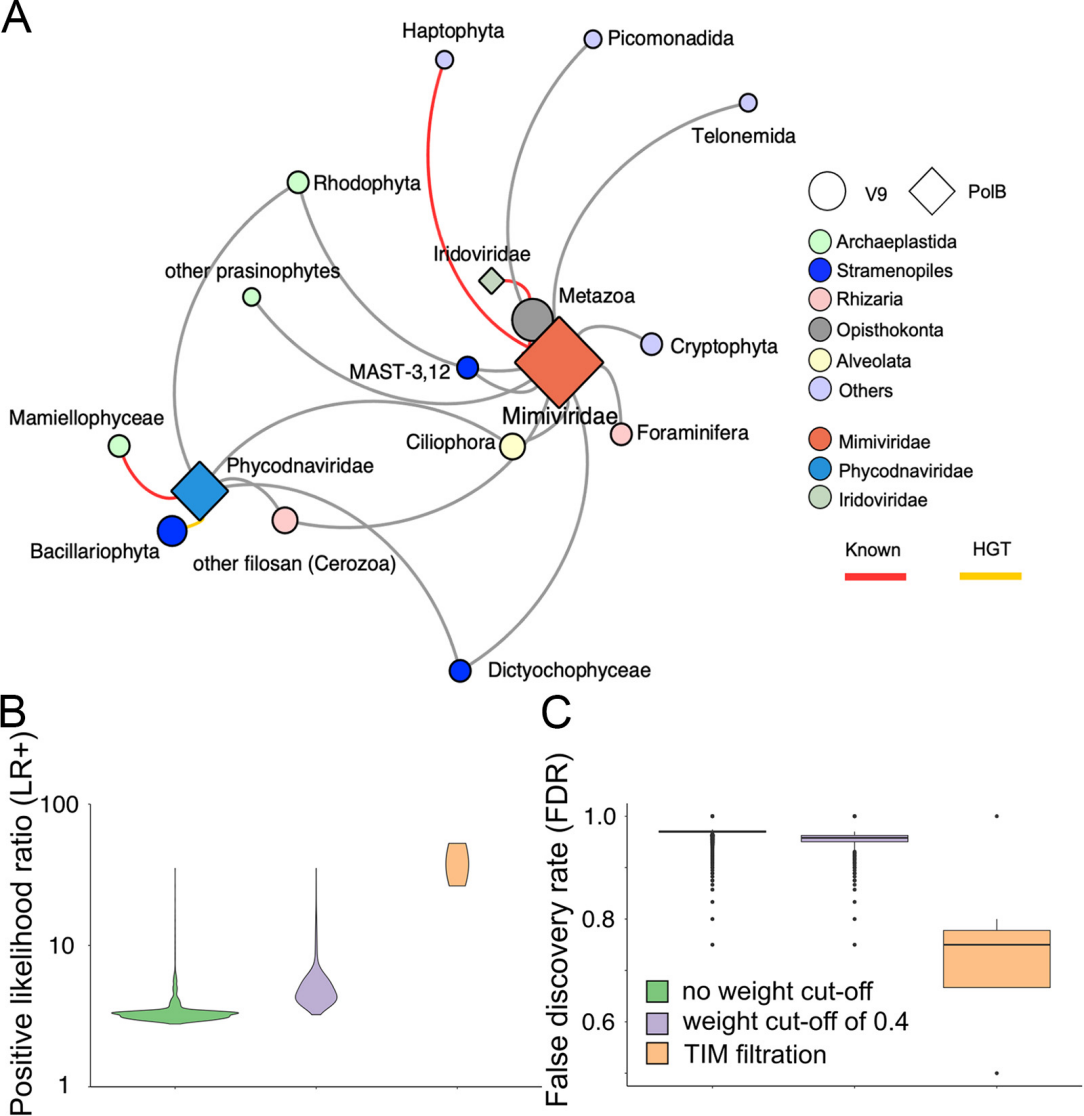
Prediction of NCLDV virus-host relationships with TIM. (A) Undirected network that shows the relationships between NCLDVs and eukaryotes after TIM filtration. The size of each node indicates the number of predicted interactions of this group. The weight of network edges as defined by the number of tree nodes enriched in each viral family subtree to specific eukaryotic major lineages in the TIM analysis. Known virus-host relationships are highlighted in red, and the pairs found to have horizontal gene transfer are highlighted in yellow (1). (B) Performance of networks on NCLDV host prediction for original FlashWeave results without a weight cutoff, weight cutoff > 0.4, and TIM filtration, plotted by ggplot2 with a bandwidth of 2. (C) FDR of networks for NCLDV host prediction with the original FlashWeave results without a weight cutoff, weight cutoff > 0.4, and TIM filtration.

Then we used a phylogeny-guided host prediction tool, TIM, to filter *polB*-V9 associations, which is based on the assumption that evolutionarily related viruses tend to infect evolutionarily related hosts (see Materials and Methods). We identified 24 eukaryotic taxonomic groups specifically associated with NCLDVs (see Fig. S7). To compare the performance of the TIM results with the raw FlashWeave results presented above, we converted the three primary eukaryotic taxonomic ranks to their associated major lineages (see Fig. S7B), and the associations were plotted as a network (Fig. 5A). This network showed that three out of nine NCLDV families (*Mimiviridae*, *Phycodnaviridae*, and *Iridoviridae*) had enriched connections in specific eukaryotic lineages. Among the network edges, known virus-host pairs were found, such as Haptophyta-*Mimiviridae*, Mamiellophyceae-*Phycodnaviridae*, and Metazoa-*Iridoviridae*. The associations in the TIM-filtered results showed a sharp improvement in performance from the original result with and without an edge weight cutoff. The average LR+ of TIM-enriched associations was 42.22, which was higher than the raw FlashWeave associations without a weight cutoff (3.43), with a weight cutoff 0.4 (5.20), and with a cutoff at 0.668 (14.23) (Fig. 5B; see also Fig. S7A). The FDR dropped from 0.97 (no cutoff) and 0.95 (weight cutoff 0.4) to 0.74 (Fig. 5C).

10.1128/mSphere.01298-20.7 FIG S7 Filtration of FlashWeave results. (A) Process of filtration. The number of retained *polB*-V9 pairs are given by *n*, we also compared the performance between TIM filtration and a further weight cutoff (0.668 was used because it provided the same number of *polB*-V9 pairs as TIM). The process used for making the final filtration and prediction is shown in yellow. (B) Phylogenetic position of NCLDVs and corresponding TIM-based predicted eukaryotic host groups. The phylogenetic tree was constructed from 501 *Tara* Oceans PolB protein sequences. Predicted hosts were shown with colored circles and annotated by NCBI taxonomies (order, class, and phylum). Download FIG S7, TIF file, 0.6 MB. Copyright © 2021 Meng et al. 2021 Meng et al. https://creativecommons.org/licenses/by/4.0/ This content is distributed under the terms of the Creative Commons Attribution 4.0 International license.

From the network, diverse putative hosts (13 lineages) emerged for *Mimiviridae*, including algae, protozoans, and metazoans. Metazoa had the most enriched nodes connected to *Mimiviridae*; additionally, MAST-3,12, Cryptophyta, Foraminifera, and Ciliophora had strong relationships with *Mimiviridae*. For *Phycodnaviridae*, there were six eukaryotic lineages retained after TIM filtration. Among these, Bacillariophyta, “other filosan (part of filosan Cercozoa),” and Mamiellophyceae had comparatively strong associations. Moreover, Rhodophyta, Ciliophora, and Dictyochophyceae had links to both *Mimiviridae* and *Phycodnaviridae*. There was also a connection between *Iridoviridae* and Metazoa.

### Associations between virophages and NCLDVs.

**FIG 6:**
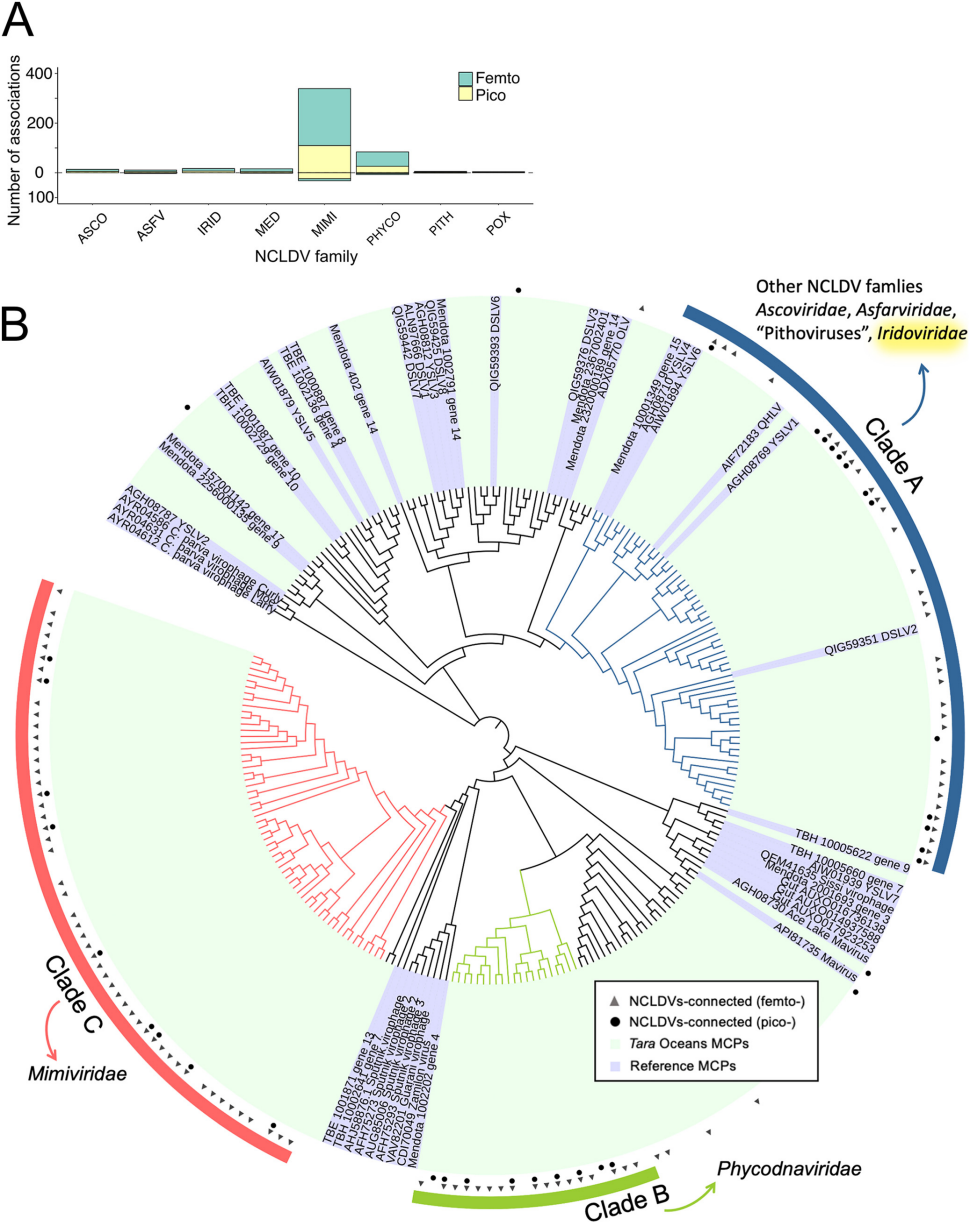
Associations between virophages and NCLDVs. (A) Number of associations with virophages is shown for seven NCLDV families and two unclassified groups, “Medusaviruses” and “Pithoviruses.” Associations in the femto-size fraction network are shown in yellow and in the pico-size fraction network are shown in green. The number of positive associations is above the zero axis, and the number of negative associations is below the zero axis. (B) Phylogenetic tree was constructed from 195 environmental virophages and 47 reference MCP sequences. The outside layer indicates three major virophage clades. The inner two layers indicate that the virophage OTUs have at least one association with NCLDVs in femto- or pico-size fraction networks.

Using 6,818 NCLDV *polB* OTUs and 195 virophage major capsid proteins (MCPs), we identified 535 FlashWeave associations (196 and 339 for pico- and femto-size fractions, respectively) (Fig. 6A). Most of the associations had positive weights (*n *=* *490), whereas some had negative weights (*n *=* *45). The average number of associations per virophage MCP was 3.2 in femto- and 5.6 in pico-size fractions. The network revealed that *Mimiviridae* had the largest number of virophage associations in both size fractions. We also detected 84 positive associations between virophages and *Phycodnaviridae*.

The phylogenetic tree defined three main virophage clades that have many connections to NCLDVs. To investigate significant relationships, a Fisher exact test was performed between virophage clades and NCLDV families. Individual families other than *Phycodnaviridae* and *Mimiviridae* did not show significance. Therefore, we used “Other NCLDVs” to include all families except *Phycodnaviridae* and *Mimiviridae*. First, we only used FlashWeave results with a weight > 0.4, as previous results showed that a FlashWeave weight of 0.4 is a suitable cutoff that produced moderate performance (Fig. 3A). From the femto-size fraction network, we found two significantly enriched connections (Fig. 6B): one was between virophage clade C and *Mimiviridae* (*P* = 0.0022), and the other was between clade A and “Other NCLDVs” (*P* = 0.0439). Another significantly enriched relationship between virophage clade B and *Phycodnaviridae* (*P* = 0.0410) was found in pico-size fractions when we used all associations without edge weight cutoff.

Finally, we examined HGTs of virophage MCPs in NCLDV genomes. We found a candidate HGT, that is, the presence of an MCP gene from virophage clade A in a metagenome-assembled genome belonging to *Iridoviridae*. This result was consistent with the Fisher exact test result, which revealed a connection between virophage clade A and the group “Other NCLDVs,” including *Iridoviridae*.

## DISCUSSION

NCLDVs can infect a wide range of eukaryotes, from unicellular to multicellular organisms (36). However, we are still far from a comprehensive knowledge of their hosts because few have been isolated so far. Therefore, better host prediction algorithms are needed to understand the ecological functions and evolutionary significance of NCLDVs. To make these predictions, we constructed global ocean co-occurrence networks based on the marine metagenome and metabarcoding data sets from 85 stations of the *Tara* Oceans expedition, which cover all major oceanic provinces across an extensive latitudinal gradient from pole to pole. The edges (associations) between *polB* and V9 nodes (OTUs) in the networks were generated using FlashWeave. The networks were particularly dense (Fig. 2A; see also Fig. S2A), thus suggesting that NCLDVs interact with numerous eukaryotes in the ocean. This was expected given the high abundance and diversity of NCLDVs in marine environments (19, 22) and the identification of HGT between these viruses and diverse eukaryotic lineages (25). The networks were dominated by the *Mimiviridae* nodes, which is consistent with previous reports that *Mimiviridae* is the most abundant and has the widest array of transcribed genes out of NCLDV families in marine environments (23, 24). *Mimiviridae* was known to infect amoebae, algae, and stramenopiles (3). In our study, these three eukaryotic groups were all found to have numerous associations with *Mimiviridae*. *Phycodnaviridae* has been known to infect many species of aquatic organisms, such as *Emiliania huxleyi* (Haptophyta), *Ectocarpus siliculosus* (Phaeophyceae), *Chlorella heliozoae* (Trebouxiophyceae), and *Ostreococcus tauri* (Mamiellophyceae) (37–39). Correspondingly, plenty of associations of *Phycodnaviridae* were found in the co-occurrence networks. For the eukaryotic nodes, all high taxonomic rank groups, including the SAR supergroup (i.e., Stramenopiles, Alveolata, and Rhizaria), Opisthokonta, Archaeplastida, Amoebozoa, Excavata, and other eukaryotes, have associations with NCLDVs. Among these groups, the SAR supergroup contributed the most (∼68%) *polB*-V9 associations. However, this is still lower than in other microbial co-occurrence analyses; for example, a previous study showed SAR supergroup dominated ∼92% of the total aquatic microbial associations (40). A substantial proportion (∼32%) of NCLDV-eukaryote interactions were from non-SAR groups, which covered the known NCLDV host range, such as Archaeplastida and Haptophyta.

However, it is difficult to accurately predict NCLDV hosts from constructed networks because of the high degree of associations per *polB* OTU (Fig. 3A). It is known that the abundance dynamics of some NCLDVs and their hosts (e.g., *Emiliania huxleyi* and Heterosigma akashiwo viruses) are sometimes not concordant (32, 33). Due to this nonlinear mechanism of virus-host interactions (e.g., viral lysis), as well as the complexity of microbial communities and external environmental drivers, many noisy signals exist in co-occurrence networks (41). In order to overcome the limitations, additional processing was usually performed in previous studies to filter the high dimensional associations to predict the meaningful interactions, such as weight cutoff or a combination of different inference methods (19, 31). Qualitatively identifying known pairs and detecting HGTs (without validation by isolation) have been commonly used to assess prediction reliability (19, 30). However, no previous study quantitatively assessed the performance of co-occurrence networks when predicting NCLDV-host relationships. Therefore, we aimed to (i) quantitatively assess the performance of co-occurrence-based host prediction for NCLDVs and (ii) improve the prediction results using filtering methods.

In a previous study of bacteriophage host prediction, ROC curves were used as an assessment metric to compare different prediction methods (42). However, the number of known virus-host pairs of NCLDVs is not sufficient to generate a data set for ROC assessment. Therefore, in this study, we carried out an alternative method, the LR+, to assess the performance. LR+ is calculated with two relative values, sensitivity and specificity (Fig. 1). The LR+ of co-occurrence-based host predictions for positive associations was higher than 1 and increased along with increasing cutoff values for the edge weights (Fig. 4A). These LR+ values indicate that FlashWeave can increase the probability of predicting true positives (43). Our result demonstrated that the co-occurrence-based host prediction of NCLDVs outperformed random prediction (i.e., random inference of virus-host pairs).

We also found that true positive predictions only existed in positive weight associations, whereas negative weight associations did not contribute to NCLDV-host detection (see Fig. S4B). This result indicates that the abundance dynamics of NCLDVs and their potential hosts were positively correlated with each other in the analyzed samples, which were collected at a global scale; this might be because NCLDVs detected in the data set were active viruses that replicate locally in their hosts. Similar results were obtained in other co-occurrence-based host prediction studies (30, 44). However, several experimental studies showed that the abundance dynamics of NCLDVs and hosts showed a delay in time (32, 33). It is possible that the global-scale samples did not have sufficiently high resolution to detect negative correlations (or correlations with a time delay) due to lack of time-resolution (45). Therefore, further studies, especially those that focus on a high temporal resolution, are needed to better understand the detailed dynamics of virus-host associations and the capacity of co-occurrence-based methods for host prediction.

The networks of different size fractions showed different performance patterns in predicting NCLDV-host relationships (Fig. 4B). This pattern is not dependent on the diversity of eukaryotic communities (see Fig. S1D and E). Generally, small-sized fractions (0.8- to 5-μm and 5- to 20-μm) networks performed better than large-sized fractions (20- to 180-μm and 180- to 2,000-μm) networks. To date, most of the known NCLDV hosts are small, such as the genera *Micromonas*, *Aureococcus*, and *Prymnesium*. Because of this, our assessment method might be biased toward small size fractions as smaller organisms tend to be more abundant in the environment (46). However, it is also possible that NCLDV infections are more prevalent in smaller size fractions. This could be the reason that large-sized networks do not have LR+ ratios as great as small-sized networks.

NCLDVs also infect large organisms and can be detected as being transcriptionally active in large-sized marine samples (24). However, the relationships between *Mimiviridae* and Metazoa, the pair that have the most associations in large-sized (20- to 180-μm and 180- to 2,000-μm) networks, are still little known. Only one reference sequence was used in our assessment (47). Thus, whether the associations between free NCLDV particles and large-sized organisms are false, indirect, or true is hard to assess currently. Notably, the 0.8–inf-μm size fraction network, which covered all four individual size fractions, performed best. This might be because NCLDVs can infect not only small hosts but also hosts from a broad size range.

Trimming of low-abundance OTUs was recommended to improve the prediction of true interactions and was often used in co-occurrence studies (48, 49). In our study, however, we did not achieve such performance improvement by treating input abundance data with a rigorous filtration (upper quartile) (see Fig. S6A). This result might be because the true-positive and false-positive rates defined in this study were too low; therefore, the validation may not be sufficiently sensitive to reflect the change between different abundance trimming strategies. However, it is also possible that low-abundance NCLDV OTUs are indeed network participants, as was demonstrated in a study showing that rare cyanobacterial species might play fundamental roles in blooming (50). Our result also revealed that FlashWeave-S was better than FlashWeave-HE at predicting NCLDV-host interactions (see Fig. S6C). The difference between FlashWeave-HE and FlashWeave-S is that HE mode can remove structural zeros during network inference. Structural zero is a typical property of heterogeneous data sets, like *Tara* Oceans data sets, and may lead to false-positive edges (51). Conversely, our results suggested that retaining structural zeros did not negatively influence the result, which indicates that the “presence-absence” pattern is as informative as the “more-less” pattern when identifying NCLDV-host relationships. This result is consistent with a previous “K-r-strategist” hypothesis: some NCLDVs, like mimiviruses, are K-strategists that decay slowly and can form stable associations with their hosts (52, 53). A recent report supported these non-“boom and bust” dynamics of prasinoviruses and their hosts with an experiment-based mathematical model (54). Overall, our results support co-occurrence networks as a useful method for predicting NCLDV-host interactions in marine metagenomes and likelihood ratios as useful quantitative metrics for assessing the performance of co-occurrence analysis for viral host predictions.

Although the results generated by FlashWeave were shown to improve the accuracy of predictions, the FDR of co-occurrence was at a high level regardless of the weight cutoff (see Fig. S4A). Such a high FDR in co-occurrence networks demonstrates that condition positive connections (i.e., known interactions) are embedded in an immense pool of condition negative connections. To further refine the raw networks using FlashWeave, we developed TIM to reduce the noisy associations and improve NCLDV host prediction (35). The results showed that NCLDVs had enriched connections with 15 major eukaryotic lineages, which included 24 taxonomic groups in three different ranks (order, class, and phylum) (Fig. 5A; see also Fig. S7). Using the LR+ as a prediction diagnostic metric, NCLDV host prediction improved 12-fold with TIM filtration (Fig. 5B). FDR dropped below 23% after TIM treatment (Fig. 5C). Taken together, our results suggest the phylogeny-guided filtering method can improve the performance of co-occurrence networks in predicting the hosts of NCLDVs.

In TIM-enriched connections, some are known NCLDV-host pairs, such as *Phycodnaviridae* and Mamiellophyceae, *Mimiviridae* and Haptophyta, and *Iridoviridae* and Metazoa. Some other studies revealed that *Mimiviridae* could exclusively infect diverse putative hosts (25, 55). Our results support the assumption that *Mimiviridae* has connections with 13 eukaryotic lineages out of 15 total lineages. Among these lineages, *Mimiviridae* had the most numerous links to Metazoa. Some mimiviruses (namaoviruses) are known to infect freshwater sturgeon, *Acipenser fulvescens* (47). Metazoans are presumed to be susceptible to mimiviruses, because the choanoflagellates, a group of eukaryotes that is phylogenetically close to metazoans, were recently identified to be the host of a species of *Mimiviridae* (16). Moreover, the TIM result revealed that *Phycodnaviridae* is closely connected to Bacillariophyta, which consists of three NCBI taxonomic groups: Thalassiophysales, Cymbellales, and Bacillariophyceae. Thalassiophysales was shown to have many HGT candidates with a large range of NCLDVs, and Bacillariophyceae also has a significant HGT candidate with phaeoviruses (25). Although Dictyochophyceae itself has not been proven to be a phycodnavirus host, its sister group Pelagophyceae was experimentally identified as an AaV host (56). In addition, it is interesting to note the connection between Metazoa (Calanoida) and *Iridoviridae*. Calanoida is an order of arthropods commonly found as zooplankton; most of the sizes are 500 to 2,000 μm. The viruses of the family *Iridoviridae* infect many Arthropod species, including insects and crustaceans (18).

Furthermore, we also inferred associations between virophages and NCLDVs. To date, all isolated virophages are only known to infect *Mimiviridae* (57). As expected, *Mimiviridae* was the family with dominant connections to virophages (Fig. 6A), demonstrating the effectiveness of our approach. Recently, *in silico* evidence demonstrated that virophages can infect *Phycodnaviridae*, which indicated that the virophage host range might be larger than we know (58). In support of this hypothesis, a relatively large number of virophage OTUs were found to be associated with *Phycodnaviridae* in our study. The enrichment analysis also revealed significant connections between three virophage clades and NCLDV families (Fig. 6B). To support the enrichment analysis, we conducted an HGT analysis because gene transfers have previously been found between Sputnik virophages and giant viruses (59). Although it is difficult to completely exclude the possibility of contamination in MAGs (i.e., contamination of a virophage genome in a NCLDV MAG), our result implied a previously undescribed infectious relationship between virophage clade A and *Iridoviridae*. Overall, the results of virophage-NCLDV associations support our previous statement that co-occurrence networks inference and analysis are appropriate for investigating NCLDV interactions in marine metagenomic data.

Currently, abundance-based predictions have many limitations, including the complexity of ecological interactions (e.g., predator-prey, parasitic, and symbiotic), environmental drivers, and measurement noise (41). The limitations were shown by a high FDR in our work. Also, the high FDR comes from the scarcity of reference knowledge on virus-host interactions, which led to a small number of condition positive connections (i.e., known interactions) embedded in an immense pool of condition negative connections. Of note, these condition negative connections can correspond to either unidentified (i.e., currently unknown true interactions), indirect (e.g., through interactions between hosts and other eukaryotes), or false relationships. This unassessed part will be one of our future study directions. Apart from experiment-based approaches, homology search effectively expands the host range of NCLDVs (25, 42). The discovery of widespread insertions (endogenous viral elements) demonstrates the evolutionary impacts of viral insertions in host genomes and sheds light on identifying the species-specific NCLDV-host relationships (29, 60, 61). Our study demonstrated that FlashWeave co-occurrence inference is better than random predictions for NCLDVs and was further improved by introducing a new concept of TIM. Therefore, co-occurrence-based approaches can be used for the generation of hypotheses to be validated experimentally. The combination of co-occurrence and other approaches will lead to a better understating of the mysterious NCLDV world.

## MATERIALS AND METHODS

### Metagenomic and metabarcoding data.

The microbial metagenomic and eukaryotic metabarcoding data used in this study were previously generated from plankton samples collected by the *Tara* Oceans expedition from 2008 to 2013 (62, 63). Because our research requires paired metagenomic and metabarcoding data sets, we used data derived from the euphotic zone samples, namely, those from the surface (SRF) and Deep Chlorophyll Maximum (DCM) layers (64). Type B DNA polymerase (*polB*) was used as the marker gene for NCLDVs. A total of 6,818 NCLDV *polB* OTUs were extracted from the metagenomic data sets (i.e., the second version of the Ocean Microbial Reference Gene catalog, OM-RGC.v2) using the pplacer phylogenetic placement method (ML tree) (23, 65, 66). These *polB* sequences were classified into seven NCLDV families (*Mimiviridae*, *Phycodnaviridae*, *Marseilleviridae*, *Ascoviridae*, *Iridoviridae*, *Asfarviridae*, and *Poxviridae*) and two other giant virus groups (“Medusaviridae” and “Pithoviridae”). For eukaryotes, we used the metabarcoding data for eukaryotes, which target the 18S rRNA gene hypervariable V9 region (V9) (64). Taxonomic annotation of the eukaryotic metabarcoding data were previously performed by the *Tara* Oceans consortium using an extensive V9_PR2 reference database (64), which was derived from the original Protist Ribosomal Reference (PR2) database (67). The diversity index of eukaryotic communities was calculated using the package “vegan” (68).

### Data processing.

A relative abundance matrix for the NCLDV *polB* OTUs was extracted from OM-RGC.v2 for the samples derived from the pico-size fractions (0.22 to 1.6 μm or 0.22 to 3.0 μm). We converted the relative abundances of *polB* OTUs back to absolute read counts based on gene length and read length (assumed to be 100 nucleotides). This process was required because small decimal numbers cannot be used by FlashWeave and because relative abundance data suffer from apparent correlations, which reduce the specificity of co-occurrence networks in revealing microbial interactions (48). To build comprehensive interaction networks involving eukaryotes of different sizes, we extracted the V9 read count matrices from the metabarcoding data set for the following five size fractions: 0.8 to 5 μm; 5 to 20 μm and 3 to 20 μm (here referred to as “5 to 20 μm” for simplicity); 20 to 180 μm; 180 to 2,000 μm; and ≥0.8 μm (here referred to as “0.8–inf μm”). To create the input files for network inference, the *polB* matrix was combined with each of the V9 matrices (corresponding to different size fractions), and only the samples represented by both *polB* and V9 files were placed in new files. In total, samples from 84 *Tara* Oceans stations (a total of 560 samples for two depths and five size fractions) widely distributed across oceans were used in this study (see Fig. S1A). Depending on the individual size fractions, 84 to 127 samples were retained and included in the co-occurrence analysis (see Fig. S1B). Read counts in the newly generated matrices were normalized using centered log-ratio (*clr*) transformation after adding a pseudo count of one to all matrix elements because zero cannot be transformed in *clr.* Following *clr* normalization, we filtered out low-abundance OTUs with a lower quartile (Q1) filtering approach. Specifically, OTUs were retained in the matrices when their *clr*-normalized abundance was higher than Q1 (among the non-zero counts in the original count matrix prior to the addition of a pseudo count of one) in at least five samples. Normalization and filtering were separately applied to *polB* and V9. The numbers of OTUs in the final matrices are provided in Fig. S1C.

### Co-occurrence-based network inference.

Network inference was performed using FlashWeave (v0.15.0 [51]). FlashWeave is a fast and compositionally robust tool for ecological network inference. FlashWeave starts by performing a locally optimal Markov blanket search in order to infer all direct associations between OTUs, then connects these individual neighborhoods to form a “global” network through a combinator rule. Meta-variables (such as environmental parameters) can be included in the FlashWeave network to remove potential indirect associations. We used temperature, salinity, nitrate, phosphate, and silicate concentrations as meta-variables in our network inferences to determine their correlations with *polB* OTUs. FlashWeave provides a heterogeneous mode (FlashWeave-HE), which helps overcome sample heterogeneity. However, FlashWeave-HE may not be appropriate for the *Tara* Oceans data because it was shown to predict an insufficient number of known planktonic interactions (51). Therefore, we mainly used FlashWeave-S with default settings except for the FlashWeave normalization step and comparison between FlashWeave-S and FlashWeave-HE. A threshold to determine the statistical significance was set to alpha < 0.01. All detected pairwise associations were assigned a value called “weight” that ranged between −1 and +1. Edges with weights > 0 or < 0 were referred to as positive and negative associations, respectively. To compare the performance of FlashWeave-S to other co-occurrence methods, we used FlashWeave-HE, Spearman, and FastSpar (69). The FlashWeave-HE settings were the same as FlashWeave-S but with a command “heterogeneous.” For Spearman, we used stats.spearmanr in package “Scipy” (70). In FastSpar, we used 50 iterations, 20 excluded iterations, and a threshold of 0.1 to generate associations. To reduce the high dimensionality of the data sets, upper quartile (Q3) filtered matrices were used for comparison among FlashWeave-S, Spearman, and FastSpar.

### Network validation.

We validated the virus-host associations in inferred networks based on a confusion matrix defined by the known NCLDV-host information (Fig. 1). Briefly, we manually compiled 69 known virus-host relationships for NCLDVs (see Table S2). In the validation process, eukaryotic taxonomic groups were annotated at the level of the “Major lineages” in the extensive PR2 database (updated after publication) (64). The “Major lineages” were used in the present study because (i) the deficiency of known virus-host relationships limited the use of lower eukaryotic taxonomy ranks, such as genus, for assessment and (ii) these lineages adequately represented marine eukaryotes by covering the full spectrum of cataloged eukaryotic V9 diversity at a comparable phylogenetic depth (64). We then performed BLASTp (2.10.1 [71]) searches from the *Tara* Oceans PolB sequences against the NCLDV reference database to define groups of metagenomic PolBs with a threshold of 65% sequence identity by retaining only the best hit for each environmental PolB sequence. This threshold was determined because, by using reference PolB sequences and RefSeq protein sequence databases, we found that 60 to 70% of sequence identity could distinguish whether the NCLDVs infected hosts of the same major lineages; this was mainly tested for *Phycodnaviridae* because of the lack of host information for closely related viruses in other NCLDV families (see Table S3). Then, 65% was chosen because it could provide a better LR+ (as described below) than 60 and 70%.

10.1128/mSphere.01298-20.9 TABLE S2 The 69 reference NCLDVs used in this study. Download Table S2, DOCX file, 0.2 MB. Copyright © 2021 Meng et al. 2021 Meng et al. https://creativecommons.org/licenses/by/4.0/ This content is distributed under the terms of the Creative Commons Attribution 4.0 International license.

10.1128/mSphere.01298-20.10 TABLE S3 Threshold determination of environmental NCLDV grouping. Download Table S3, DOCX file, 0.09 MB. Copyright © 2021 Meng et al. 2021 Meng et al. https://creativecommons.org/licenses/by/4.0/ This content is distributed under the terms of the Creative Commons Attribution 4.0 International license.

The positive likelihood ratio was used in for assessment to estimate the predictions accuracy. This approach is commonly used in diagnostic testing to assess whether a test (host prediction in this study) usefully changes the probability of the existence of condition positive (true positive). In this study, the LR+ was used because host prediction is a test to discover condition positive states (72). LR+ is calculated by dividing the true-positive rate (sensitivity) by the false-positive rate (1 –specificity). If LR+ is close to 1, the performance of the prediction is comparable to a random prediction. If LR+ ≫ 1, a positive prediction result is more likely to be a true positive than that based on random prediction. From the set of detected associations between a given *polB* OTU and V9 OTUs that belong to a given major eukaryotic lineage, we only kept the best positive or negative associations (i.e., the edges with the highest absolute weights) to simplify the prediction scheme. As an auxiliary assessment, the FDR was also calculated by dividing the number of false positives by the number of positive predictions (Fig. 1). For the comparison among five size fractions, we only used the abundance in the overlap samples of 0.8- to 5-μm, 5- to 20-μm, 20- to 180-μm, and 180- to 2,000-μm sizes. So, the numbers of samples in five size fractions are comparable (*n* = 84, 88, 88, 88, and 88), which could reduce the bias that may influence the topology of networks (73).

### Phylogeny-guided filtering of host predictions and its assessment.

We developed Taxon Interaction Mapper (TIM) to improve host predictions by co-occurrence approaches (35). TIM assumes that evolutionarily related viruses tend to infect evolutionarily related hosts (18, 74) and extract the most likely virus-host associations from the co-occurrence networks. In the case that multiple and distantly related viruses infect one host (10), TIM can map the associations of one eukaryotic group to many viral branches. TIM requires a phylogenetic tree of viruses (based on marker genes) and a set of connections between viruses and eukaryotes (co-occurrence edges), and then tests whether leaves (i.e., viral OTUs) under a node of the virus tree is enriched with a specific predicted host group compared to the rest of the tree using the Fisher exact test and Benjamini-Hochberg adjustment (see Fig. S7A) (35). TIM is available from https://github.com/RomainBlancMathieu/TIM.

We pooled network associations using FlashWeave analysis for five size fractions. To build a concise and credible viral phylogenetic tree, we removed all of the PolB sequences that were absent in the FlashWeave network associations, and the remaining sequences were filtered by the amino acid sequence length (≥500 amino acids). Protein alignment was conducted using MAFFT-*linsi* (version 7.471 [75]), and 18 sequences were manually removed because they were not well aligned with other PolB sequences. A total of 501 PolB sequences were used to make a maximum likelihood phylogenetic tree with FastTree (version 2.1.11 [76]). Then, the PolB-V9 associations were mapped on the tree to calculate the significance of the enrichment of specific associations using TIM. TIM provides a list of nodes in the viral tree and associated NCBI taxonomies (order, class, and phylum) of eukaryotes that show significant enrichment in the leaves under the nodes. The TIM result was visualized with iTOL (version 5 [77]). The TIM result was converted to a network, in which nodes correspond to the major eukaryotic lineages. The network edge weight was defined by the number of tree nodes in each viral family subtree enriched with a specific major eukaryotic lineage. The network was visualized with Cytoscape [version 3.7.1] using prefuse force directed layout (78). To assess the effectiveness of TIM in improving prediction, we extracted all the associations predicted by TIM and compared their performance with the raw and weight cutoff results.

### Virophage-NCLDV associations.

We inferred the networks between NCLDVs and virophages using *mcp* as the marker gene for virophages. First, 47 reference MCP amino acid sequences were collected from public databases and used to build an HMM profile. The HMM profile was used to search against the amino acid sequences of OM-RGC v2 using HMMER hmmsearch [version 3.3.1] with the threshold of E value < 1E−90 (79). This threshold was determined by searching reference sequences against the GenomeNet nr-aa database. The search detected 195 *Tara* Oceans virophage MCP sequences in the OM-RGC database. Together with 47 reference MCPs, a phylogenetic tree of MCP amino acid sequences was built using MAFFT and FastTree.

We extracted the abundance profiles for the 195 MCP sequences from the pico-size (0.22- to 1.6-μm or 0.22- to 3.0-μm) and femto-size (<0.22-μm) fractions. We used samples from the SRF and DCM depths. PolB and MCP abundance profiles were merged into two matrices corresponding to the two virophage size fractions. Then, network inference was conducted using the FlashWeave default settings after Q1 filtration. We did not apply a quantitative assessment of NCLDV-virophage associations due to the limitation of the known NCLDV-virophage pairs and reference genomes of isolated virophages. In the MCP phylogenetic tree, three virophage clades contributed most of the NCLDV connections. Thus, an NCLDV enrichment analysis for the three clades was carried out using a Fisher exact test, and the *P* value was adjusted by the Benjamin-Hochberg method. This approach was the same as TIM, but we did not use the TIM software because the current version of TIM requires inputs of eukaryotic nodes with NCBI taxonomy annotations.

We used another approach, HGT, to predict the virophage-NCLDV interactions. First, we generated an NCLDV genome database, which includes 56 reference NCLDV genomes corresponding to our *polB* data set and 2,074 metagenome-assembled genomes from a previous study (25). A total of 827,548 coding sequences were included in this database. Then, 195 virophage MCPs from the metagenomic data were BLASTp searched against this database using an E value cutoff of 1E−10 (with a minimum query coverage of 50% and a minimum sequence identity of 50%). If a virophage MCP obtained a hit in the NCLDV genome database with a lower E value compared to hits in the MCP database (the hit to itself was removed), the hit in the NCLDV genome database was considered an HGT candidate.

### Data availability.

Processed frequency data and associations used in assessment are available from GenomeNet (https://www.genome.jp/ftp/db/community/tara/Cooccurrence/).
