# Supplementary figures and images for: Quantitative Assessment of Nucleocytoplasmic Large DNA Virus and Host Interactions Predicted by Co-occurrence Analyses
Source: mSphere. 2021 Apr 21;6(2):e01298-20. doi: 10.1128/mSphere.01298-20 (PMC8546719; doi:10.1128/mSphere.01298-20)

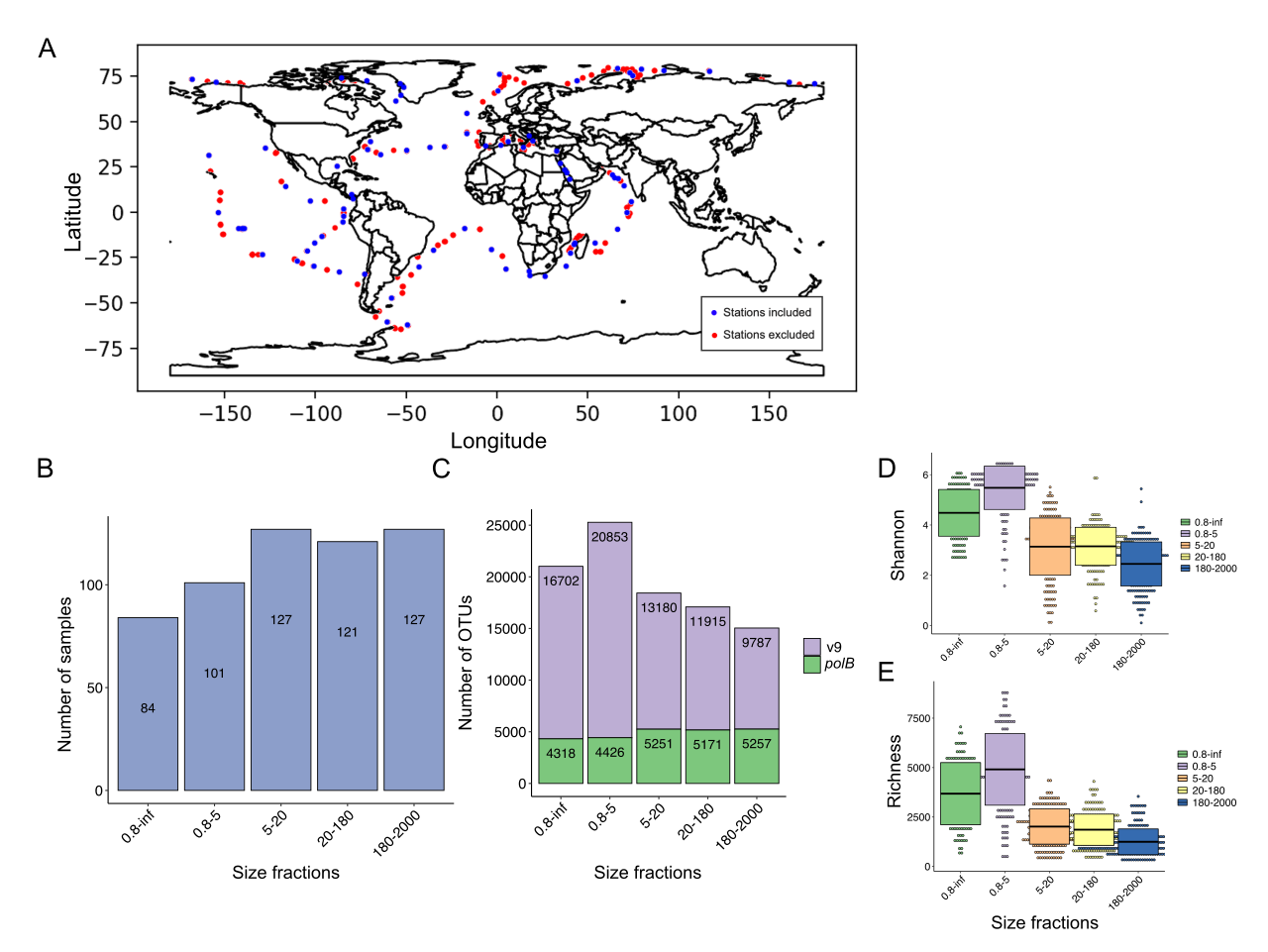

Supplement: FIG S1 [file msphere.01298-20-sf001.tif]

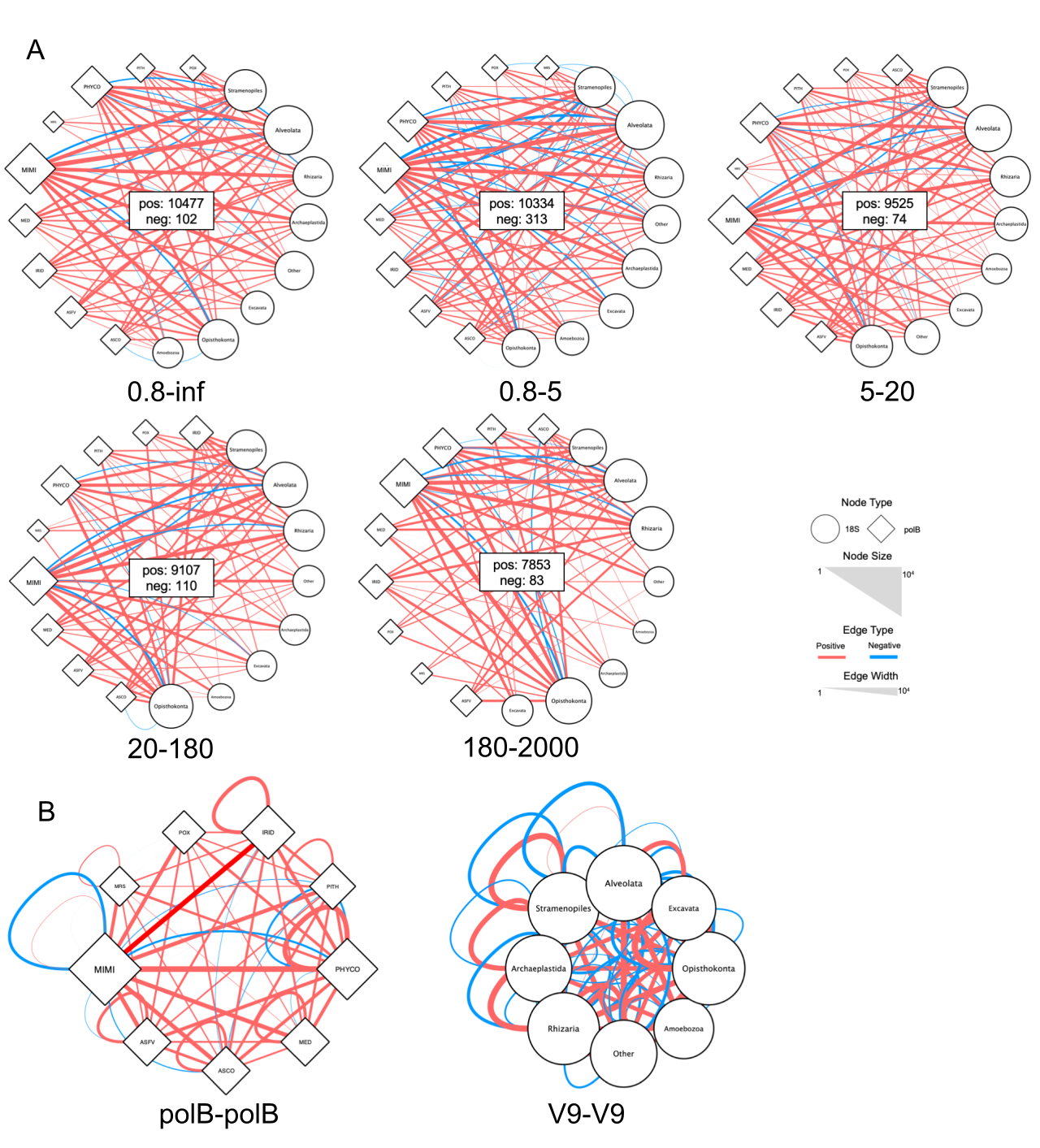

Supplement: FIG S2 [file msphere.01298-20-sf002.tif]

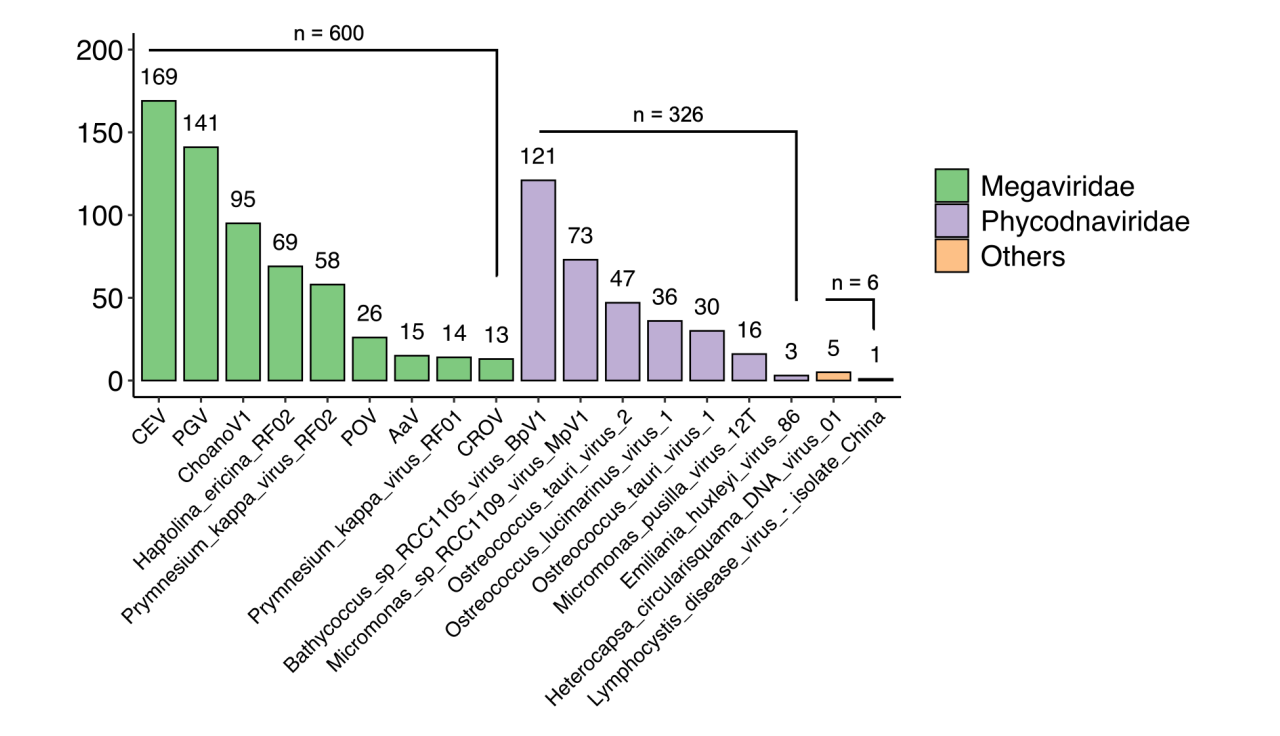

Supplement: FIG S3 [file msphere.01298-20-sf003.tif]

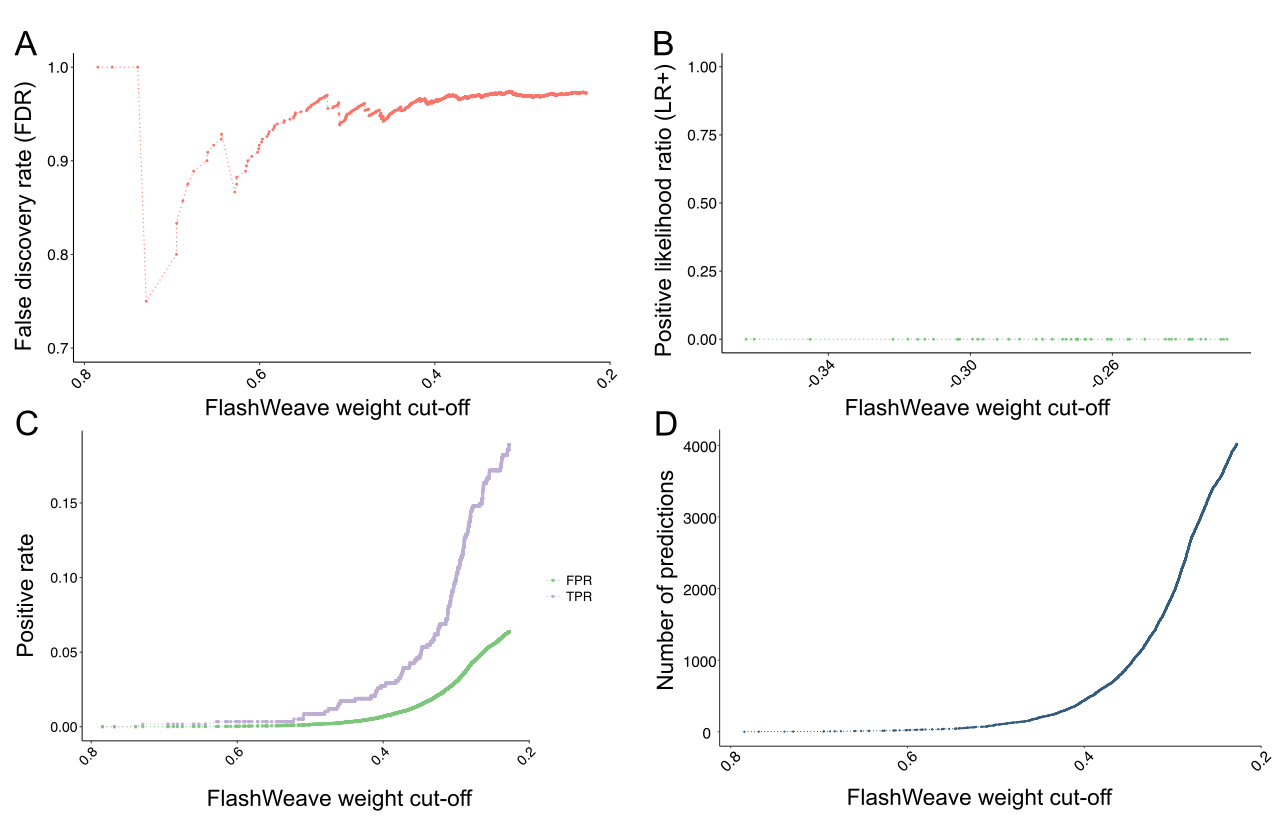

Supplement: FIG S4 [file msphere.01298-20-sf004.tif]

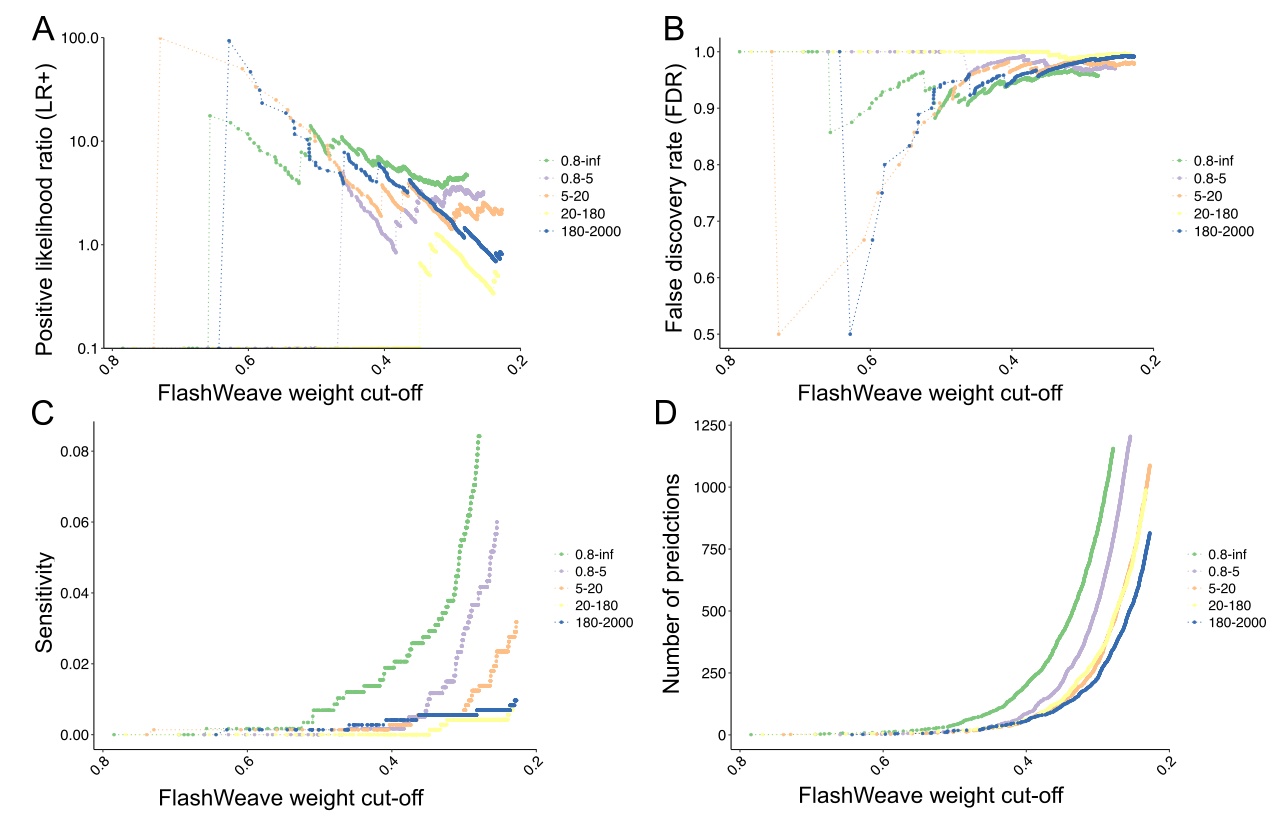

Supplement: FIG S5 [file msphere.01298-20-sf005.tif]

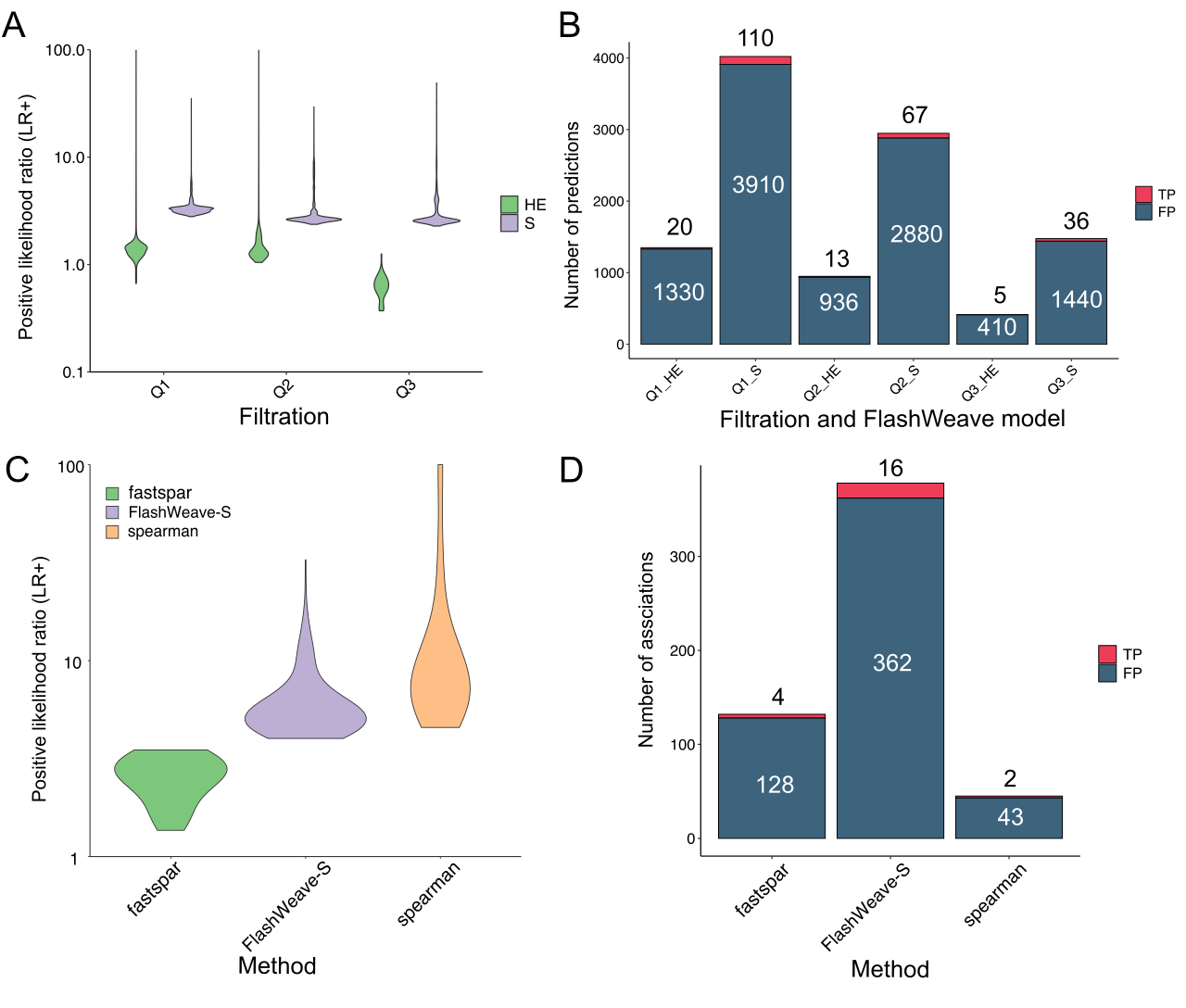

Supplement: FIG S6 [file msphere.01298-20-sf006.tif]

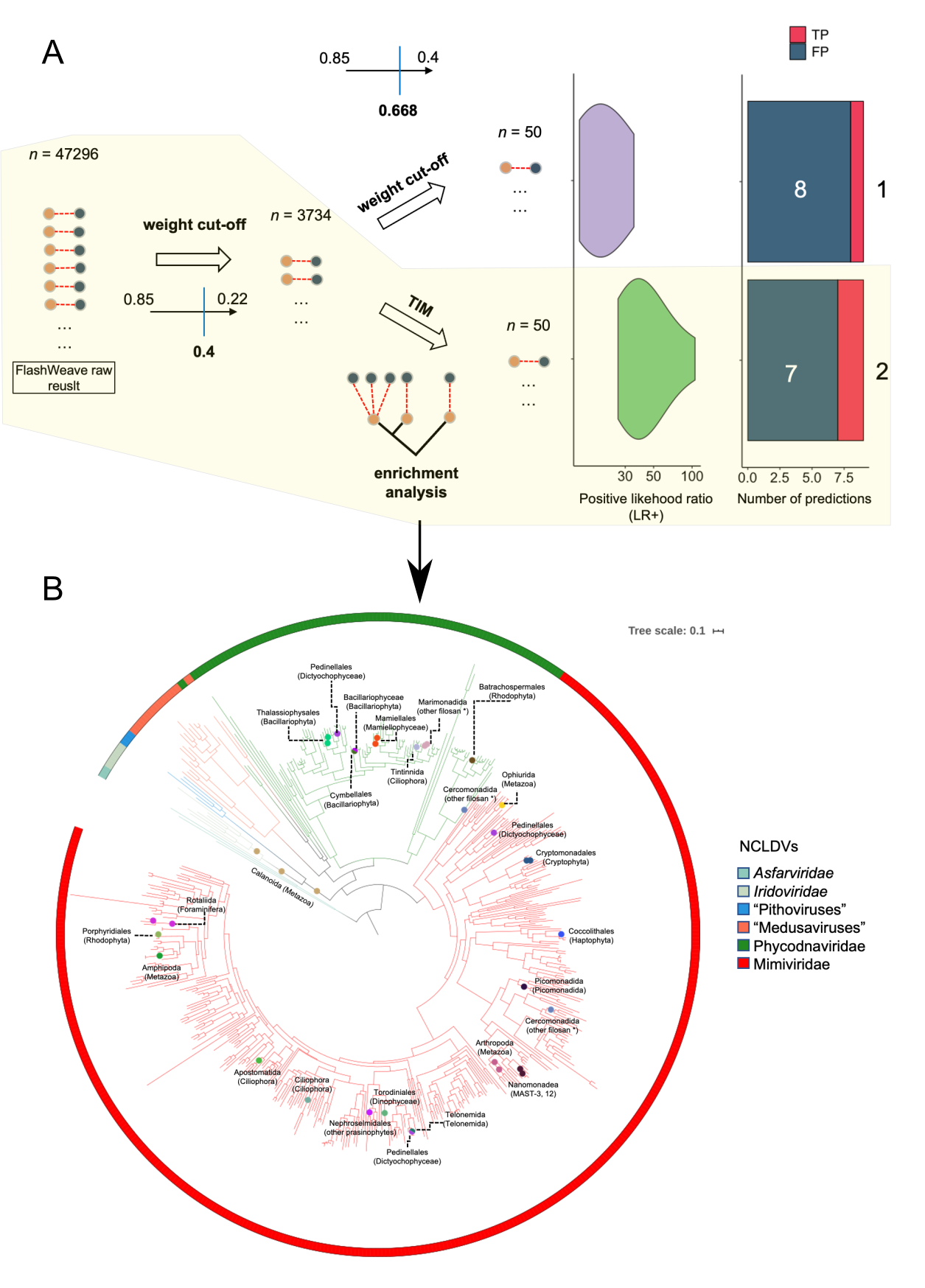

Supplement: FIG S7 [file msphere.01298-20-sf007.tif]
