# Supplementary material for: Quantitative Assessment of Nucleocytoplasmic Large DNA Virus and Host Interactions Predicted by Co-occurrence Analyses
Source: mSphere. 2021 Apr 21;6(2):e01298-20. doi: 10.1128/mSphere.01298-20 (PMC8546719; doi:10.1128/mSphere.01298-20)
Supplement: Supplementary file 2 [file msphere.01298-20-st001.docx]

Size_fractions	PolB	Family	Envrionmental_parameter
0.8-5	TARA_138_DCM_0.22-3_G_C18424242_1_gene501927	Ascoviridae	NO2
0.8-inf	TARA_180_SRF_lt-0.22_G_scaffold9939_5_gene48951	Ascoviridae	Salinity
20-180	TARA_206_MES_lt-0.22_G_scaffold78978_1_gene218156	Ascoviridae	Temperature
0.8-5	TARA_155_DCM_0.22-3_G_C16978172_1_gene567032	Medusavirus	NO2
20-180	TARA_007_SRF_0.22-1.6_G_scaffold79565_1_gene70629	Megaviridae	Temperature
5-20	TARA_009_SRF_0.22-1.6_G_scaffold107825_1_gene135836	Megaviridae	Temperature
5-20	TARA_031_SRF_0.22-1.6_G_C28711937_1_gene471690	Megaviridae	Salinity
20-180	TARA_038_DCM_0.22-1.6_G_C23589122_1_gene515547	Megaviridae	NO2
180-2000	TARA_042_SRF_0.22-1.6_G_scaffold239468_1_gene192167	Megaviridae	Temperature
180-2000	TARA_076_SRF_0.22-0.45_G_scaffold67722_1_gene45184	Megaviridae	Temperature
180-2000	TARA_084_SRF_0.22-3_G_scaffold217252_1_gene156539	Megaviridae	PO4
5-20	TARA_085_DCM_0.22-3_G_scaffold263925_1_gene243728	Megaviridae	Si
0.8-5	TARA_085_SRF_0.22-3_G_C16197045_1_gene301689	Megaviridae	Temperature
180-2000	TARA_085_SRF_0.22-3_G_scaffold143071_1_gene112591	Megaviridae	Si
0.8-inf	TARA_102_DCM_0.22-3_G_scaffold400037_1_gene474877	Megaviridae	NO2
180-2000	TARA_111_SRF_0.22-3_G_scaffold275213_1_gene259640	Megaviridae	NO2
20-180	TARA_122_DCM_0.22-0.45_G_C14241023_1_gene864905	Megaviridae	Temperature
180-2000	TARA_125_MIX_0.22-0.45_G_scaffold296071_1_gene285960	Megaviridae	Temperature
0.8-5	TARA_133_SRF_0.22-3_G_C26534727_1_gene887577	Megaviridae	Salinity
180-2000	TARA_158_SRF_0.22-3_G_scaffold245594_1_gene258773	Megaviridae	Temperature
5-20	TARA_163_SRF_0.22-3_G_scaffold123902_2_gene136645	Megaviridae	Temperature
180-2000	TARA_082_DCM_0.22-3_G_scaffold104031_1_gene99802	Phycodnaviridae	Temperature
180-2000	TARA_193_SRF_0.22-3_G_scaffold165463_1_gene212660	Phycodnaviridae	Salinity
5-20	TARA_194_DCM_lt-0.22_G_scaffold15132_1_gene46748	Phycodnaviridae	Temperature
0.8-5	TARA_140_SRF_0.22-3_G_C20955321_1_gene443592	Pithoviridae	Salinity
