# Supplementary material for: Quantitative Assessment of Nucleocytoplasmic Large DNA Virus and Host Interactions Predicted by Co-occurrence Analyses
Source: mSphere. 2021 Apr 21;6(2):e01298-20. doi: 10.1128/mSphere.01298-20 (PMC8546719; doi:10.1128/mSphere.01298-20)
Supplement: Supplementary file 3 [file msphere.01298-20-st002.docx]

Virus	Class	Order	Family	Genus	Species	Major_lineage
**MIMI_ChoanoV1**	Unknown	Choanoflagellatea	Unknown	Unknown	Unknown	Choanoflagellatea
**MIMI_CEV**	Haptophyta	Prymnesiales	Prymnesiaceae	Haptolina	ericina	Haptophyta
**MIMI_Haptolina_ericina_RF02**	Haptophyta	Prymnesiales	Prymnesiaceae	Haptolina	ericina	Haptophyta
**MIMI_PGV**	Haptophyta	Phaeocystales	Phaeocystaceae	Phaeocystis	globosa	Haptophyta
**MIMI_Prymnesium_kappa_virus_RF02**	Haptophyta	Prymnesiales	Prymnesiaceae	Prymnesium	kappa	Haptophyta
**MIMI_TetV-1**	Chlorodendrophyceae	Chlorodendrales	Chlorodendraceae	Tetraselmis	Unknown	other core chlorophyte
**MIMI_Prymnesium_kappa_virus_RF01**	Haptophyta	Prymnesiales	Prymnesiaceae	Prymnesium	kappa	Haptophyta
**MIMI_AaV**	Pelagophyceae	Pelagomonadales	Pelagomonadaceae	Aureococcus	anophagefferens	Pelagophyceae
**MIMI_CROV**	Unknown	Bicoecea	Cafeteriaceae	Cafeteria	roenbergensis	Bicoecea
**PHYCO_Ostreococcus_lucimarinus_virus_1**	Mamiellophyceae	Mamiellales	Bathycoccaceae	Ostreococcus	lucimarinus	Mamiellophyceae
**PHYCO_Ostreococcus_tauri_virus_1**	Mamiellophyceae	Mamiellales	Bathycoccaceae	Ostreococcus	tauri	Mamiellophyceae
**PHYCO_Ostreococcus_tauri_virus_2**	Mamiellophyceae	Mamiellales	Bathycoccaceae	Ostreococcus	tauri	Mamiellophyceae
**PHYCO_Micromonas_sp_RCC1109_virus_MpV1**	Mamiellophyceae	Mamiellales	Mamiellaceae	Micromonas	pusilla	Mamiellophyceae
**PHYCO_Micromonas_pusilla_virus_12T**	Mamiellophyceae	Mamiellales	Mamiellaceae	Micromonas	pusilla	Mamiellophyceae
**PHYCO_Bathycoccus_sp_RCC1105_virus_BpV1**	Mamiellophyceae	Mamiellales	Bathycoccaceae	Bathycoccus	prasinos	Mamiellophyceae
**PHYCO_Paramecium_bursaria_Chlorella_virus_FR483**	Trebouxiophyceae	Chlorellales	Chlorellaceae	Micractinium	conductrix	Trebouxiophyceae
**PHYCO_Acanthocystis_turfacea_Chlorella_virus_1**	Trebouxiophyceae	Chlorellales	Chlorellaceae	Chlorella	heliozoae	Trebouxiophyceae
**PHYCO_Paramecium_bursaria_Chlorella_virus_1**	Trebouxiophyceae	Chlorellales	Chlorellaceae	Chlorella	Unknown	Trebouxiophyceae
**PHYCO_Feldmannia_species_virus**	Phaeophyceae	Ectocarpales	Acinetosporaceae	Feldmannia	Unknown	Phaeophyceae
**PHYCO_Ectocarpus_siliculosus_virus_1**	Phaeophyceae	Ectocarpalesn	Ectocarpaceae	Ectocarpus	siliculosus	Phaeophyceae
**PHYCO_Emiliania_huxleyi_virus_86**	Phaeophyceae	Isochrysidales	Noelaerhabdaceae	Emiliania	huxleyi	Haptophyta
**PHYCO_Pandoravirus_salinus**	Unknown	Centramoebida	Acanthamoebidae	Acanthamoeba	castellanii	other Lobosa
**PHYCO_Pandoravirus_dulcis**	Unknown	Centramoebida	Acanthamoebidae	Acanthamoeba	castellanii	other Lobosa
**PHYCO_Heterosigma_akashiwo_virus_01**	Raphidophyceae	Chattonellales	Chattonellaceae	Heterosigma	akashiwo	Raphidophyceae
**MIMI_OLPV1**	Unknown	Unknown	Unknown	Unknown	Unknown	Unknown
**MIMI_OLPV2**	Unknown	Unknown	Unknown	Unknown	Unknown	Unknown
**MIMI_POV**	Unknown	Unknown	Unknown	Unknown	Unknown	Unknown
**MIMI_megavirus_chiliensis**	Unknown	Centramoebida	Acanthamoebidae	Acanthamoeba	castellanii	other Lobosa
**MIMI_megavirus_lba**	Unknown	Unknown	Amoebidae	Amoeba	Unknown	Tubulinea
**MIMI_megavirus_courdo7**	Unknown	Unknown	Amoebidae	Amoeba	Unknown	Tubulinea
**MIMI_moumouvirus_ochan**	Unknown	Centramoebida	Acanthamoebidae	Acanthamoeba	polyphaga	other Lobosa
**MIMI_moumouvirus_monve**	Unknown	Centramoebida	Acanthamoebidae	Acanthamoeba	polyphaga	other Lobosa
**MIMI_moumouvirus_M10A**	Unknown	Centramoebida	Acanthamoebidae	Acanthamoeba	polyphaga	other Lobosa
**MIMI_terra2**	Unknown	Centramoebida	Acanthamoebidae	Acanthamoeba	polyphaga	other Lobosa
**MIMI_apmv**	Unknown	Centramoebida	Acanthamoebidae	Acanthamoeba	polyphaga	other Lobosa
**MIMI_hirudovirus_Sangsue**	Unknown	Centramoebida	Acanthamoebidae	Acanthamoeba	polyphaga	other Lobosa
**MIMI_pointerouge1**	Unknown	Centramoebida	Acanthamoebidae	Acanthamoeba	polyphaga	other Lobosa
**MIMI_Tupanvirus_soda_lake**	Unknown	Unknown	Hartmannellidae	Vermamoeba	Unknown	Tubulinea
**MIMI_Tupanvirus_deep_ocean**	Unknown	Centramoebida	Acanthamoebidae	Acanthamoeba	castellanii	other Lobosa
**MIMI_Klosneuvirus_KNV1_1**	Unknown	Unknown	Unknown	Unknown	Unknown	Unknown
**MIMI_Namao_virus**	Actinopteri	Acipenseriformes	Acipenseridae	Acipenser	fulvescens	Metazoa
**MED_Medusavirus_Unagene_392**	Unknown	Centramoebida	Acanthamoebidae	Acanthamoeba	castellanii	other Lobosa
**MIMI_Klosneuvirus_KNV1_2**	Unknown	Unknown	Unknown	Unknown	Unknown	Unknown
**MRS_lausannevirus**	Unknown	Centramoebida	Acanthamoebidae	Acanthamoeba	Unknown	other Lobosa
**MRS_T19**	Unknown	Centramoebida	Acanthamoebidae	Acanthamoeba	Unknown	other Lobosa
**ASCO_Heliothis_virescens_ascovirus_3e**	Insecta	Lepidoptera	Noctuidae	Heliothis	Unknown	Metazoa
**ASCO_Spodoptera_frugiperda_ascovirus_1a**	Insecta	Lepidoptera	Noctuidae	Spodoptera	Unknown	Metazoa
**ASCO_Trichoplusia_ni_ascovirus_2c**	Insecta	Lepidoptera	Noctuidae	Trichoplusia	Unknown	Metazoa
**IRID_Infectious_spleen_and_kidney_necrosis_virus**	Actinopteri	Syngnathiformes	Callionymidae	Synchiropus	Unknown	Metazoa
**IRID_Lymphocystis_disease_virus_-_isolate_China**	Actinopteri	Centrarchiformes	Centrarchoidei	Unknown	Unknown	Metazoa
**IRID_Lymphocystis_disease_virus_1**	Actinopteri	Centrarchiformes	Centrarchoidei	Unknown	Unknown	Metazoa
**IRID_Singapore_grouper_iridovirus**	Actinopteri	Perciformes	Serranidae	Epinephelus	Unknown	Metazoa
**IRID_Invertebrate_iridescent_virus_3**	Insecta	Diptera	Culicidae	Psorophora	Unknown	Metazoa
**IRID_Invertebrate_iridescent_virus_6**	Insecta	Orthoptera	Gryllidae	Gryllus	Unknown	Metazoa
**PITH_pithovirus**	Unknown	Centramoebida	Acanthamoebidae	Acanthamoeba	polyphaga	other Lobosa
**POX_Amsacta_moorei_entomopoxvirus_L**	Insecta	Lepidoptera	Lymantriidae	Lymantria	Unknown	Metazoa
**POX_Melanoplus_sanguinipes_entomopoxvirus**	Insecta	Orthoptera	Acrididae	Locusta	Unknown	Metazoa
**POX_Deerpox_virus_W-1170-84**	Mammalia	Cetartiodactyla	cervidae	Odocoileus	Unknown	Metazoa
**POX_Yaba-like_disease_virus**	Mammalia	Primates	Hominidae	Homo	Unknown	Metazoa
**POX_Monkeypox_virus_Zaire-96-I-16**	Mammalia	Primates	Hominidae	Homo	Unknown	Metazoa
**POX_Molluscum_contagiosum_virus_subtype_1**	Mammalia	Primates	Hominidae	Homo	Unknown	Metazoa
**POX_Orf_virus**	Mammalia	Primates	Hominidae	Homo	Unknown	Metazoa
**POX_Fowlpox_virus**	Aves	Galliformes	Phasianidae	Meleagris	Unknown	Metazoa
**POX_Nile_crocodilepox_virus**	Unknown	Crocodylia	Crocodylidae	Crocodylus	Unknown	Metazoa
**ASFV_African_swine_fever_virus**	Mammalia	Cetartiodactyla	Suidae	Sus	Unknown	Metazoa
**ASFV_Pacmanvirus_A23**	Unknown	Centramoebida	Acanthamoebidae	Acanthamoeba	Unknown	other Lobosa
**ASFV_Heterocapsa_circularisquama_DNA_virus_01**	Dinophyceae	Peridiniales	Heterocapsaceae	Heterocapsa	Unknown	Dinophyceae
**MIMI_Bodo_saltans_virus**	Unknown	Kinetoplastida	Bodonidae	Bodo	Unknown	Kinetoplastida

Notes:

1.Cells filled with green:
 Viruses infect non-Amoeba protists

2.Major_lineage:
 85 deep-branching eukaryotic lineages represented in our global oceans metabarcodingdata set
 Reference:
 https://www.ncbi.nlm.nih.gov/pubmed/25999516

3.Some viruses, for example IRID_Lymphocystis_disease_virus_1, could infect more than one host species. But their hosts, large size metazoan animals, are not included in *Tara*
only list one of their known hosts, more information could be found by reference links.
