# Supplementary material for: Quantitative Assessment of Nucleocytoplasmic Large DNA Virus and Host Interactions Predicted by Co-occurrence Analyses
Source: mSphere. 2021 Apr 21;6(2):e01298-20. doi: 10.1128/mSphere.01298-20 (PMC8546719; doi:10.1128/mSphere.01298-20)
Supplement: Supplementary file 4 [file msphere.01298-20-st003.docx]

Accession	The best hit with different host major lineage	Identity	Major Lineage	Accession
NULL	Acanthamoeba polyphaga mimivirus	46.5	Unknown	YP_003986825.1
NULL	Acanthamoeba polyphaga mimivirus	40.47	Unknown	YP_003986825.1
NULL	Bombus impatiens	34.55	Unknown	XP_003484917.1
NULL	Cafeteria roenbergensis virus BV-PW1	34	Bicoecea	YP_003970130.1
YP_009173620.1	Entamoeba invadens IP1	34.65	Unknown	XP_004185906.1
NULL	MIMI_POV	37.81	Pyramimonadales	NULL
YP_009173620.1	Cafeteria roenbergensis virus BV-PW1	36.76	Bicoecea	YP_003970130.1
NULL	Acanthamoeba polyphaga mimivirus	45.63	other Lobosa	YP_003986825.1
YP_003970130.1	Megavirus chiliensis	34.67	other Lobosa	YP_004894633.1
YP_007676285.1	Dishui lake phycodnavirus 1	59.6	Unknown	YP_009465906.1
YP_004061614.1	Dishui lake phycodnavirus 1	58.1	Unknown	YP_009465906.1
YP_007676285.1	Dishui lake phycodnavirus 1	57.65	Unknown	YP_009465906.1
YP_007676285.1	Dishui lake phycodnavirus 1	59.21	Unknown	YP_009465906.1
YP_004061614.1	Dishui lake phycodnavirus 1	56.99	Unknown	YP_009465906.1
YP_009325633.1	Yellowstone lake phycodnavirus 1	38.68	Unknown	YP_009174732.1
YP_001427279.1	Yellowstone lake phycodnavirus 1	39.04	Unknown	YP_009174732.1
YP_001427279.1	Bathycoccus sp. RCC1105 virus BpV1	40.16	Mamiellophyceae	YP_004061614.1
NP_077578.1	Chlamydomonas reinhardtii	34.57	Unknown	XP_001689909.1
YP_009665694.1	Aedes aegypti	32.28	Unknown	XP_021705412.1
YP_293784.1	Agaricus bisporus var. burnettii JB137-S8	37.81	Unknown	XP_007327213.1
YP_009507574.1	Yellowstone lake phycodnavirus 2	40.34	Unknown	YP_009174650.1
YP_009507841.1	Rhizophagus irregularis DAOM 181602=DAOM 197198	26.03	Unknown	XP_025181784.1
NULL	Megavirus chiliensis	35.27	other Lobosa	YP_004894633.1
NULL	MIMI_Prymnesium_kappa_virus_RF01	45.7	Haptophyta	NULL
